# Bis(Dicarbollide) Complexes of Transition Metals: How Substituents in Dicarbollide Ligands Affect the Geometry and Properties of the Complexes

**DOI:** 10.3390/molecules29153510

**Published:** 2024-07-26

**Authors:** Igor B. Sivaev

**Affiliations:** A. N. Nesmeyanov Institute of Organoelement Compounds, 28 Vavilov Str., Moscow 119991, Russia; sivaev@ineos.ac.ru

**Keywords:** bis(dicarbollide) complexes, intramolecular hydrogen bonds, rotation conformers, biological activity, molecular switches

## Abstract

The interaction between different types of substituents in dicarbollide ligands and their influence on the stabilization of various rotational conformers (rotamers) of transition metal bis(dicarbollide) complexes [3,3′-M(1,2-C_2_B_9_H_11_)_2_]^−^ are considered. It has been shown that the formation of intramolecular CH···X hydrogen bonds between dicarbollide ligands is determined by the size of the proton acceptor atom X rather than its electronegativity. Due to the stabilization of rotamers with different dipole moments, intramolecular hydrogen bonds between ligands in transition metal bis(dicarbollide) complexes can have a significant impact on the biological properties of their derivatives. In the presence of external complexing metals, weak intramolecular CH···X hydrogen bonds can be broken to form stronger X—>M donor-acceptor bonds. This process is accompanied by the mutual rotation of dicarbollide ligands and can be used in sensors and molecular switches based on transition metal bis(dicarbollide) complexes.

## 1. Introduction

When the bis(dicarbollide) complexes of iron and cobalt [3,3′-M(1,2-C_2_B_9_H_11_)_2_]^−^ (M = Fe, Co) were first synthesized in the mid-1960s, they were immediately recognized as analogs of the corresponding metallocenes [1,2], which was later confirmed by their extraordinary stability and ability to undergo hydrogen substitution reactions by various atoms and groups [3,4,5,6,7,8]. Later, a large number of sandwich and semi-sandwich dicarbollide complexes of other transition metals were synthesized [9,10] and some of them have found applications in a wide variety of fields, from catalysis [11,12,13,14,15,16,17,18,19] and material science [20,21,22,23,24] to medicine [25,26,27,28,29,30,31,32,33,34,35,36,37].

Despite a certain similarity between cyclopentadienide (C_5_H_5_)^−^ and dicarbollide (1,2-C_2_B_9_H_11_)^2−^ ligands, there are a number of differences between them, which leads to a difference in the properties of transition metal complexes based on those ligands. The first and most obvious difference is the larger charge of the dicarbollide ligand. This can be successfully overcome by introducing the so-called charge-compensate substituents of the onium type [38,39]. Another distinguishing feature of the dicarbollide ligand is its greater donor capacity, which leads to more efficient stabilization of the higher oxidation states of transition metals compared with the cyclopentadienide ligand [40]. For example, the replacement of one cyclopentadienide ligand in ferrocene by a dicarbollide one leads to a decrease in the redox potential of Fe^2+^/Fe^3+^ to −0.43 V for [3-Cp-3,1,2-FeC_2_B_9_H_11_], and the replacement of the second one, to −0.78 V for [3,3′-Fe(1,2-C_2_B_9_H_11_)_2_]^−^ relative to Fc/Fc^+^ [41]. The difference in the electron-donating capacity of the cyclopentadienyl and dicarbollide ligands can be partially compensated by introducing electron-ithdrawing substituents into the latter [42,43,44,45]. Nevertheless, it is the anionic form characteristic of bis(dicarbollide) complexes that is not typical for metallocene complexes [46].

The most stable of the metallocenes are the 18-e ferrocene FeCp_2_, ruthenocene RuCp_2_, and osmocene OsCp_2_ since in each of them, the metal reaches the electron configuration of an inert gas. This also explains the easy oxidation of cobaltocene to cobalticinium ion [CoCp_2_]^+^, which has remarkable stability. Likewise, the most stable of the transition metal bis(dicarbollide) complexes is the 18-e cobalt(III) bis(dicarbollide) [3,3′-Co(1,2-C_2_B_9_H_11_)_2_]^−^ [3]. It is stable toward strong mineral acids, whereas heating in 40% aqueous sodium hydroxide results in partial degradation of the dicarbollide ligand rather than destruction of the complex [47]. The 17-e iron(III) bis(dicarbollide) [3,3′-Fe(1,2-C_2_B_9_H_11_)_2_]^−^ is also stable toward acids, but some of its *C*-substituted derivatives are destroyed by alkali [48] or other transition metals [49]. The formation of sandwich bis(dicarbollide) complexes is very common for both cobalt and iron, with only a few examples of half-sandwich dicarbollide complexes of cobalt and nickel known [50,51]. At the same time, both 19-e and 18-e sandwich nickel bis(dicarbollide) complexes, [3,3′-Ni(1,2-C_2_B_9_H_11_)_2_]^−^ and [3,3′-Ni(1,2-C_2_B_9_H_11_)_2_], are less stable [52] and, upon reactions with 2,2′-bipyridine [53,54,55,56,57], 1,10-phenanthroline [57,58] or triphenylphosphine [59] give the corresponding half-sandwich dicarbollide complexes, while the reaction with pyridine leads to partial degradation and rearrangement of one of the dicarbollide ligands [60]. Sandwich bis(dicarbollide) complexes of ruthenium, rhodium, palladium, and iridium are also known, but the formation of half-sandwich dicarbollide complexes is more typical for these metals [9].

In addition to the differences associated with the donor ability of cyclopentadienide and dicarbollide ligands and the electronic structure of sandwich complexes based on them, there are a number of differences due to the structure of the ligands themselves. In contrast to cyclopentadienide ligands, hydrogen atoms in the pentagonal face of the dicarbollide ligand are not located in the ligand plane but are directed from the center of the icosahedron, decreasing the ligand cone angle. Unlike the previous ones, this difference is determined by the geometry of the dicarbollide ligand and cannot be compensated in any way. Moreover, the substitution of hydrogens by other atoms or groups leads to an even greater decrease in the ligand cone angle. This is most noticeable in bis(dicarbollide) complexes of transition metals where the interaction between substituents in the dicarbollide ligands becomes one of the main factors determining the properties of the complexes.

A detailed consideration of such intramolecular interactions and their effect on the properties of transition metal bis(dicarbollide) complexes is the goal of this review.

## 2. Dicarbollide vs. Cyclopentadienide: Geometry and Energy

To begin with, we would like to compare some geometrical characteristics of the sandwich cyclopentadienide and dicarbollide complexes. In ferrocene [Fe(η^5^-C_5_H_5_)_2_], the Fe^2+^ ion is sandwiched between two cyclopentadienide ligands, and the distance between the planes of the cyclopentadienide ligands is 3.3 Å. The iron(II) ion here as “atomic ball-bearing” enabling nearly free rotation of the ligands with the eclipsed conformation is slightly preferred (the rotation barrier is ~1.1 kcal/mol) [61]. In iron(II) bis(dicarbollide) complex (Me_4_N)_2_[3,3′-Fe(1,2-C_2_B_9_H_11_)_2_], the distance between the planes of the dicarbollide ligands is much shorter (2.98 Å [62]) due to their strong donating effect. This way, the ligands come closer to each other, which implies an increase in interactions at their periphery. Taking into account that the hydrogen atoms in the pentagonal face of the dicarbollide ligand are directed away from the center of the icosahedron, the hydrogen atoms of the opposite ligands experience repulsive interactions, which leads to the energetic preference for the staggered conformation. If we move from bis(dicarbollide) complexes with two carbon atoms in the ligand, which leads to the formation of a number of different rotational conformers (rotamers), to complexes with simpler pure boron ligands, such as (Bu_4_N)_3_[Cu(η^5^-B_11_H_11_)_2_] [63] with the distance between the pentagonal planes of the ligands of 3.01 Å, we see that the ligands adopt the staggered conformation due to the mutual repulsion of the hydrogen atoms of the opposite ligands (the rotation barrier is 6.9 kcal/mol [64]). Clearly, in the case of bis(dicarbollide) complexes, the staggered conformation of the dicarbolide ligands will also be preferable.

From a formal point of view, the dicarbollide ligands are formed by removing one BH vertex from icosahedral carboranes C_2_B_10_H_12_. Therefore, they include ligands derived from all three isomers of icosahedral caborane (*ortho-*, *meta-* and *para-*). However, the vast majority of bis(dicarbollide) complexes (>99%) are complexes based on the dicarbollide ligand derived from *ortho*-carborane, while the number of such complexes based on ligands derived from *meta*- and *para*-carboranes is very small [3,4,65,66].

**Figure 1:**
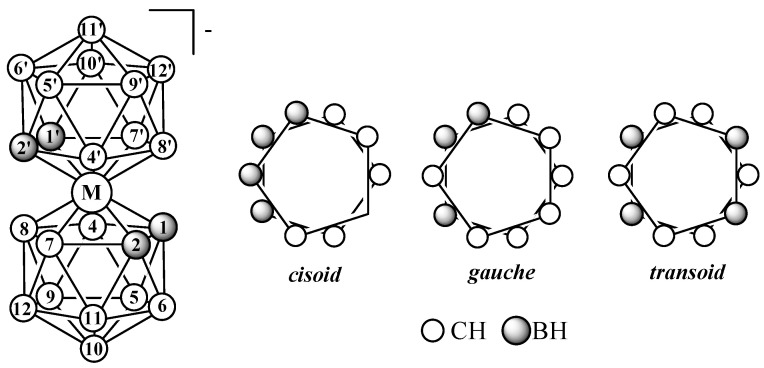
Atom numeration and possible mutual orientation of dicarbollide ligands in the transition metal bis(1,2-dicarbollide) complexes [3,3′-M(1,2-C_2_B_9_H_11_)_2_]^−^.

Therefore, in this review, only bis(dicarbollide) complexes with dicarbollide ligands derived from *ortho*-carborane containing a C–C bond in the open pentagonal face will be considered. The dicarbollide ligand contains two carbon atoms and three boron atoms in the open pentagonal face, which leads to energy nonequivalence of various rotational conformations of bis(dicarbollide) complexes, among which *cisoid*- (the ligand rotation angle is 36°), *gauche*- (the ligand rotation angle is 108°), and *transoid*- (the ligand rotation angle is 180°) can be distinguished (Figure 1).

The preference for a particular conformation depends on the nature of the metal and its oxidation state. A typical example is nickel bis(dicarbollide) complexes, which can exist in two stable states—paramagnetic nickel(III) bis(dicarbollide) [3,3′-Ni(1,2-C_2_B_9_H_11_)_2_]^−^ and diamagnetic nickel(IV) bis(dicarbollide) [3,3′-Ni(1,2-C_2_B_9_H_11_)_2_] [4]. In this case, for nickel(III) bis(dicarbollide), the *transoid* conformation is preferable, while for nickel(IV) bis(dicarbollide), the *cisoid* conformation is the most energetically favorable [20,67]. It has been suggested that the reversible rotational motion of dicarbollide ligands in nickel bis(dicarbollide) complexes can be used to create rotational molecular devices, and several such devices have been created [68,69,70].

**Figure 2:**
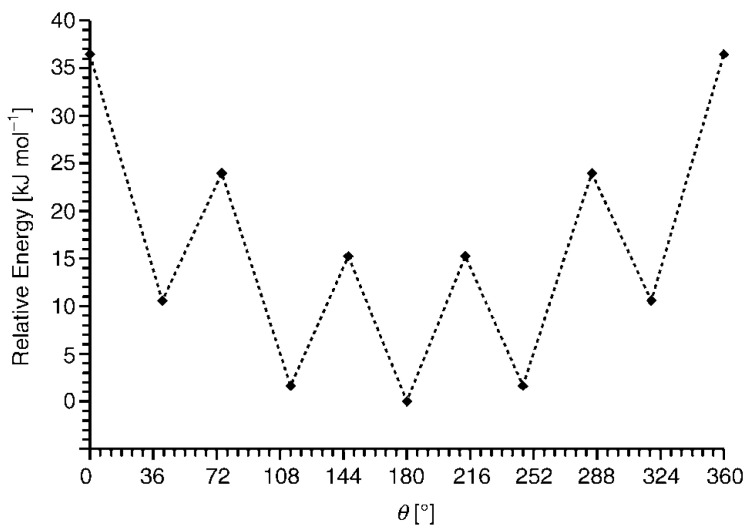
Energy profile for rotation of the dicarbollide ligands in [3,3′-Co(1,2-C_2_B_9_H_11_)_2_]^−^. Reproduced with permission from reference [71]. Copyright © (2005) John Wiley & Sons.

However, due to the relatively low stability of nickel bis(dicarbollide) complexes (see above) and the paramagnetic nature of iron bis(dicarbollide) [3,3′-Fe(1,2-C_2_B_9_H_11_)_2_]^−^, the most attention is drawn to highly stable diamagnetic cobalt bis(dicarbollide) [3,3′-Co(1,2-C_2_B_9_H_11_)_2_]^−^, whose derivatives are well identified by NMR spectroscopy [3,5,6,8]. According to quantum chemical calculations, the *transoid* conformation is the most energetically preferable for [3,3′-Co(1,2-C_2_B_9_H_11_)_2_]^−^ (Figure 2) [71]. However, according to the Cambridge Structural Database, the solid-state cobalt bis(dicarbollide) adopts the *transoid* conformation only in less than 10% of cases, while the *cisoid* conformation is found in more than 75% of cases [72].

This is due to the rather low energy barriers to the mutual rotation of the dicarbollide ligands (~3–5 kcal/mol), on the one hand, and the large difference in the dipole moments of different conformations, on the other hand. The *transoid* rotamer has a null dipole moment, which does not contribute to the formation of a strong crystal packing, while the dipole moment of the *cisoid* rotamer is 5.4 D [72]. Therefore, in inhomogeneous media including the solid state, the *cisoid* conformation with its large dipole moment is often preferred.

It is quite obvious that the replacement of hydrogen atoms in the pentagonal face of the dicarbollide ligand with other atoms or groups should lead to an increase in the barrier of mutual rotation of the ligands and, consequently, to the stabilization of certain rotamers due to intramolecular interactions between the ligands.

## 3. Halogen Derivatives of Transition Metal Bis(Dicarbollides)

**Figure 3:**
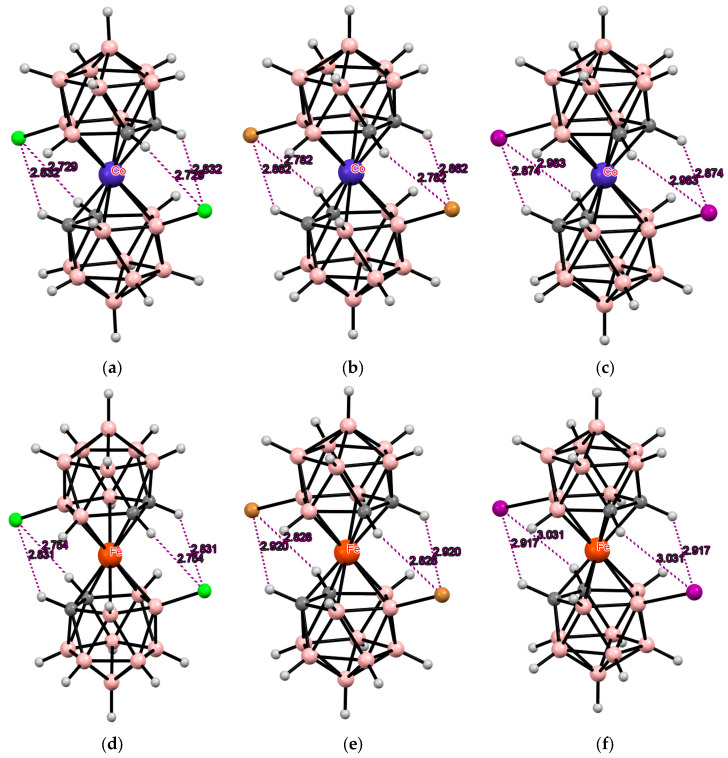
Intramolecular CH···X hydrogen bonding in the solid-state structures of [8,8′-Cl_2_-3,3′-Co(1,2-C_2_B_9_H_10_)_2_]^−^ (**a**); [8,8′-Br_2_-3,3′-Co(1,2-C_2_B_9_H_10_)_2_]^−^ (**b**); [8,8′-I_2_-3,3′-Co(1,2-C_2_B_9_H_10_)_2_]^−^ (**c**); [8,8′-Cl_2_-3,3′-Fe(1,2-C_2_B_9_H_10_)_2_]^−^ (**d**); [8,8′-Br_2_-3,3′-Fe(1,2-C_2_B_9_H_10_)_2_]^−^ (**e**); and [8,8′-I_2_-3,3′-Co(1,2-C_2_B_9_H_10_)_2_]^−^ (**f**).

The simplest example of such a modification is the electrophilic halogenation of cobalt bis(dicarbollide) (COSAN), leading to 8,8′-dihalogen derivatives [8,8′-X_2_-3,3′-Co(1,2-C_2_B_9_H_10_)_2_]^−^ (X = F, Cl, Br, I) [3,6]. This can lead to the formation of intramolecular CH···X hydrogen bonds between the halogen substituents in one ligand and the slightly acidic *CH* groups in the other ligand. Two pairs of the intramolecular CH···X hydrogen bonds are responsible for stabilization of the *transoid* conformation of the metallacarborane anion in the solid-state structures of the 8,8′-dihalogen derivatives of cobalt bis(dicarbollide) K[8,8′-Cl_2_-3,3′-Co(1,2-C_2_B_9_H_10_)_2_] [73], (PPN)[8,8′-Cl_2_-3,3′-Co(1,2-C_2_B_9_H_10_)_2_] [74], (BEDT-TTF)[8,8′-Cl_2_-3,3′-Co(1,2-C_2_B_9_H_10_)_2_] [75], (BEDT-TTF)_2_[8,8′-Cl_2_-3,3′-Co(1,2-C_2_B_9_H_10_)_2_] [76], (BMDT-TTF)_4_[8,8′-Cl_2_-3,3′-Co(1,2-C_2_B_9_H_10_)_2_] [76], (BEDT-TTF)[8,8′-Cl_1.25_Br_0.75_-3,3′-Co(1,2-C_2_B_9_H_10_)_2_] [75], (BEDT-TTF)[8,8′-Br_2_-3,3′-Co(1,2-C_2_B_9_H_10_)_2_] [77], (BEDT-TTF)_2_[8,8′-Br_2_-3,3′-Co(1,2-C_2_B_9_H_10_)_2_] [77], (BMDT-TTF)_4_[8,8′-Br_2_-3,3′-Co(1,2-C_2_B_9_H_10_)_2_] [77], Cs[8,8′-I_2_-3,3′-Co(1,2-C_2_B_9_H_10_)_2_] [78], (TTF)[8,8′-I_2_-3,3′-Co(1,2-C_2_B_9_H_10_)_2_] [79], (BEDT-TTF)_2_[8,8′-I_2_-3,3′-Co(1,2-C_2_B_9_H_10_)_2_] [79], (BMDT-TTF)_4_[8,8′-I_2_-3,3′-Co(1,2-C_2_B_9_H_10_)_2_] [79], and {(MeCN)_2_Ag [8,8′-I_2_-3,3′-Co(1,2-C_2_B_9_H_10_)_2_]}_n_ [80] (Figure 3). The same *transoid* conformation of the metallacarborane anion was found in the solid-state structures of the 8,8′-dihalogen derivatives of iron bis(dicarbollide) K[8,8′-Cl_2_-3,3′-Fe(1,2-C_2_B_9_H_10_)_2_] [73], (BEDT-TTF)_2_[8,8′-Cl_2_-3,3′-Fe(1,2-C_2_B_9_H_10_)_2_] [81], (BPDT-TTF)_2_[8,8′-Cl_2_-3,3′-Fe(1,2-C_2_B_9_H_10_)_2_] [82], (EOTT)_2_[8,8′-Cl_2_-3,3′-Fe(1,2-C_2_B_9_H_10_)_2_] [82], (BEDT-TTF)_2_[8,8′-Br_2_-3,3′-Fe(1,2-C_2_B_9_H_10_)_2_] [83], Cs[8,8′-I_2_-3,3′-Fe(1,2-C_2_B_9_H_10_)_2_]·MeCN [84], and (BEDT-TTF)[8,8′-I_2_-3,3′-Fe(1,2-C_2_B_9_H_10_)_2_] [83] (Figure 3).

According to Pauling’s principle, the strength of a hydrogen bond should increase with the increase of the electronegativity of the acceptor atom [85]; however, in the bis(dicarbollide) complexes, the van der Waals radius of a halogen seems to be more important than its electronegativity. Indeed, although the fluorine atom has the highest electronegativity, its size is insufficient for the formation of intramolecular CH···F hydrogen bonds in the 8,8′-difluoro derivative of cobalt bis(dicarbollide) (Bu_4_N)[8,8′-F_2_-3,3′-Co(1,2-C_2_B_9_H_10_)_2_] where the metallacarborane anion adopts the *cisoid* conformation [86]. Moreover, in some cases, intramolecular CH···Cl hydrogen bonds are not strong enough to stabilize the *transoid* conformation of the 8,8′-dichloro derivative of cobalt bis(dicarbollide). In particular, the *gauche* conformation was found in the solid-state structure of (TMTTF)[8,8′-Cl_2_-3,3′-Co(1,2-C_2_B_9_H_10_)_2_]_2_ [87].

According to quantum chemical calculations for the [8,8′-I_2_-3,3′-Co(1,2-C_2_B_9_H_10_)_2_]^−^ anion, the energy of the *transoid* conformation stabilized by two pairs of the intramolecular CH···I hydrogen bonds is 4.2 kcal mol^–1^ lower than the energy of the *gauche* conformation stabilized by two individual CH···I bonds and is 13.8 kcal mol^–1^ lower than the energy of the *cisoid* conformation, in which these interactions are completely absent [88].

Based on this, it can be assumed that the *transoid* conformation will be quite stable even in the case of the 8-monoiodo derivative of cobalt bis(dicarbollide). Indeed, it is the *transoid* conformation of the cobalt bis(dicarbollide) anion that was found in the crystal structures of Cs[8-I-3,3′-Co(1,2-C_2_B_9_H_10_)(1′,2′-C_2_B_9_H_11_)] [89] and (BEDT-TTF)_2_[8-I-3,3′-Co(1,2-C_2_B_9_H_10_)(1′,2′-C_2_B_9_H_11_)] [90].

Knowledge about the conformation of bis(dicarbollide) complexes is based mainly on single-crystal X-ray diffraction data. Therefore, studies that provide information on the conformation of bis(dicarbollide) complexes in solution are of particular interest.

**Figure 4:**
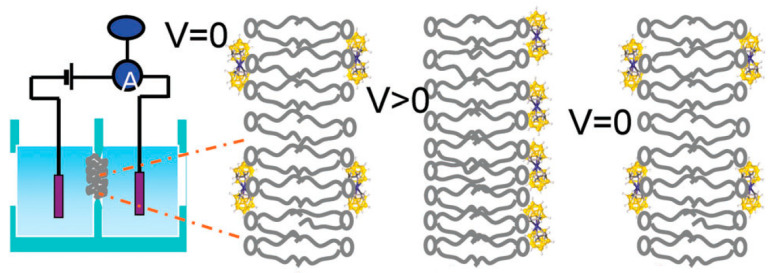
A scheme of the voltage-jump experiments with a planar lipid bilayer membrane. Reproduced with permission from reference [91] with permission of The Royal Chemical Society.

Among them is the study of transmembrane translocation (flip-flop) of cobalt bis(dicarbollide) derivatives across the lipid bilayer diphytanoylphosphatidylcholine membrane induced by the application of a voltage jump. In the absence of applied voltage, the cobalt bis(dicarbollide) anions bind symmetrically to the lipid membrane. The application of voltage leads to a transmembrane redistribution (flip) of the compounds, resulting in the relaxation of the initially high electric current to a low level due to the depletion of the compound on one of the membrane surfaces. Then, the potential is switched to zero while the current exhibits a similar relaxation but of the opposite sign. This process is a result of a return (flop) of the cobalt bis(dicarbollide) anions to the initial symmetrical distribution at two sides of the membrane (Figure 4) [91].

**Figure 5:**
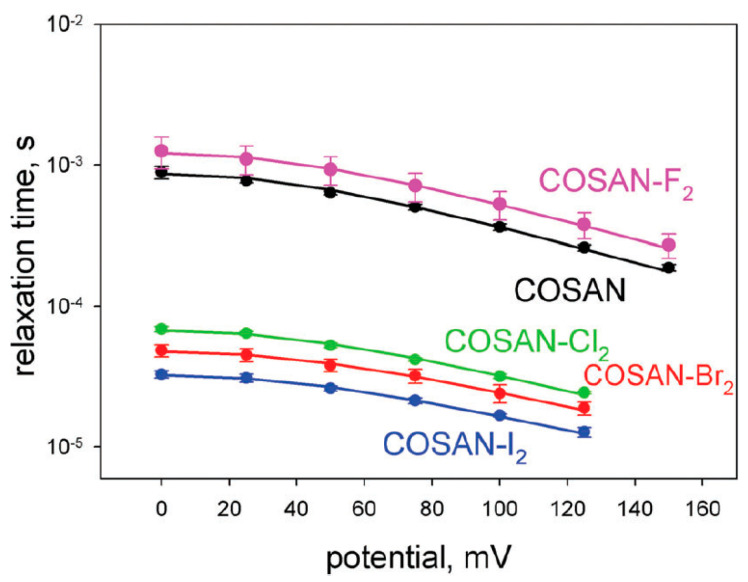
Voltage dependence of the relaxation time τ for cobalt bis(dicarbollide) and its 8,8′-dihalogen derivatives. Reproduced from reference [91] with permission of The Royal Chemical Society.

It was found that the relaxation time of 8,8′-dichloro-, 8,8′-dibromo-, and 8,8′-diiodo derivatives of cobalt bis(dicarbollide) is more than an order of magnitude shorter than that for the 8,8′-difluoro derivative and parent cobalt bis(dicarbollide) (Figure 5) [91]. Accordingly, the translocation rate for derivatives in which the *transoid* conformation with zero dipole moment is stabilized by intramolecular hydrogen bonds is much higher than for compounds where such stabilization is absent (Table 1) [91]. The slower translocation rate of the 8,8′-difluoro derivative compared with the parent cobalt bis(dicarbollide) is apparently due to its large dipole moment, which slows down the translocation.

**Figure 6:**
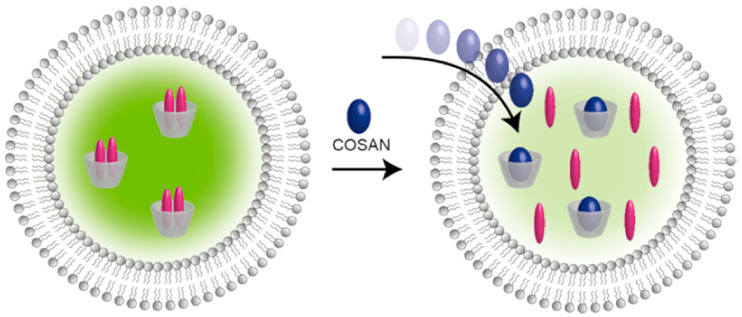
Schematic representation of the translocation of cobalt bis(dicarbollide) derivatives through a vesicular lipid bilayer monitored using γ-CD/DSS reporter pair. Reproduced with permission from reference [92]. Copyright © (2019) American Chemical Society.

In a related experiment, the kinetics of the translocation of cobalt bis(dicarbollide) and its derivatives through the POPC/POPS liposome membrane was measured by the change in fluorescence of the encapsulated complex of γ-cyclodextrin with dapoxyl sodium sulfonate, which occurs due to the translocation and the displacement of the dye by cobalt bis(dicarbollide) derivatives (Figure 6). The of 8,8′-dichloro and 8,8′-diiodo derivatives showed the fastest kinetics compared with the parent cobalt bis(dicarbollide) and other derivatives, in which there was no stabilization of the *transoid* conformation. The next two are cobalt bis(dicarbollide) derived from *meta*-carborane, [2,2′-Co(1,7-C_2_B_9_H_11_)_2_]^−^, which has a significantly smaller dipole moment than [3,3′-Co(1,2-C_2_B_9_H_11_)_2_]^−^ (2.4 D), and the 8-monoiodo derivative [8-I-3,3′-Co(1,2-C_2_B_9_H_10_)(1′,2′-C_2_B_9_H_11_)]^−^. Since the last derivative has different ligands, its *transoid* conformation cannot have a null dipole moment, but it is in the *transoid* conformation that the dipole moment is minimal [92].

However, both studies described above concern the translocation of cobalt bis(dicarbollide) and its derivatives through bilayer membranes, the surface of which is the interface between homogeneous and heterogeneous media. Therefore, of particular interest is the study of cobalt bis(dicarbollide) derivatives in solution, which is a completely homogeneous medium. ^1^H NMR spectroscopy has previously been shown to be an effective way to detect hydrogen bonding of organic solutes [93], including the formation of intramolecular hydrogen bonds in various organic compounds [94,95]. It has also been shown that ^1^H NMR spectroscopy can be successfully used to detect intramolecular CH···X hydrogen bonds in carborane derivatives [96,97].

In a comparative study of the ^1^H NMR spectra of cobalt bis(dicarbollide) and its 8,8′-dihalogen derivatives [8,8′-X_2_-3,3′-Co(1,2-C_2_B_9_H_10_)_2_]^−^ (X = H, F, Cl, Br, I) in different solvents (CDCl_3_, CD_2_Cl_2_, acetone-*d*_6_, DMSO-*d*_6_, methanol-*d*_4_), it was found that the chemical shifts of the *CH* groups of the chloro, bromo, and iodo derivatives, the structures of which are stabilized by intramolecular hydrogen bonds, are in the range of 4.21–4.46 ppm, whereas in the spectra of the parent cobalt bis(dicarbollide) and its fluoro derivative, where such bonds are absent, the signals of the *CH* groups are in the range of 3.71–3.99 ppm (Table 2). This is in complete agreement with the first criterion for hydrogen bonding, namely, a strong downfield shift of the hydrogen atom signal. This shift decreases in all solvents in the series I > Br > Cl, which is consistent with the strength of intramolecular hydrogen bonds in 8,8′-dihalogen derivatives of cobalt bis(dicarbollide) [98].

According to the second criterion for the formation of an intramolecular hydrogen bond, the difference in the chemical shifts of the hydrogen atoms in the ^1^H NMR spectra in CDCl_3_ and DMSO-*d*_6_ participating in the hydrogen bonding should be much less than the difference in the chemical shifts of the atoms not participating in the hydrogen bond. Indeed, for the derivatives with the intramolecular C-H...X hydrogen bonds (X = Cl, Br, I) this difference is negligibly small, while for the derivatives with no such bonds (X = H, F), the difference is ~0.2 ppm. These results indicate the retention of the intramolecular C-H...X hydrogen bonds and, consequently, the *transoid* conformation of the cobalt bis(dicarbollide) derivatives [8,8′-X_2_-3,3′-Co(1,2-C_2_B_9_H_10_)_2_]^−^ (X = Cl, Br, I) in solution [98].

**Figure 7:**
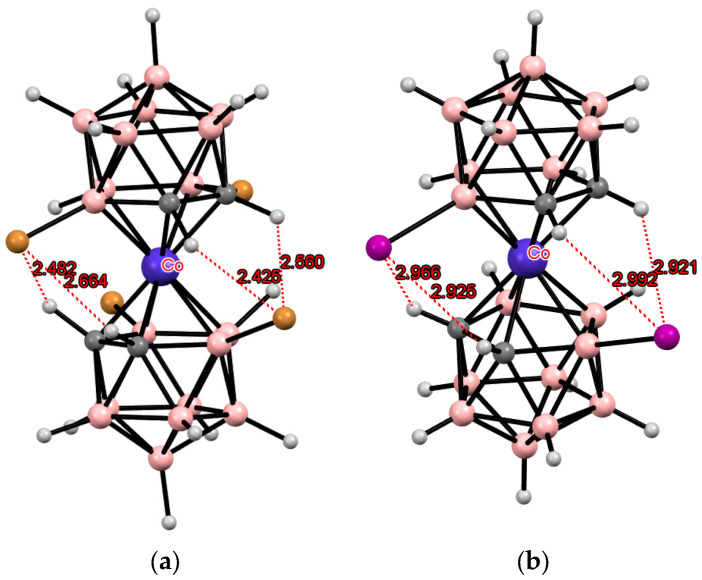
Intramolecular CH···X hydrogen bonding in the solid-state structures of [4,4′,7,7′-Br_4_-3,3′-Co(1,2-C_2_B_9_H_9_)_2_]^−^ (**a**) and [4,4′-I_2_-3,3′-Co(1,2-C_2_B_9_H_10_)_2_]^−^ (**b**) anions.

Transition metal bis(dicarbollides) with halogen substituents in other positions of the C_2_B_3_ ring have been studied to a much lesser extent, which is largely due to the use of indirect methods for their synthesis. The 4,4′,7,7′-tetrabromo derivative of cobalt bis(dicarbollide) was prepared by the reaction of CoCl_2_ with [9,11-Br_2_-*nido*-7,8-C_2_B_9_H_10_]^−^ in tetrahydrofuran and *n*-BuLi as a base. The single-crystal X-ray diffraction study of (Et_4_N)[4,4′7,7′-Br_4_-3,3′-Co(1,2-C_2_B_9_H_9_)_2_] revealed that in the solid state, the [4,4′7,7′-Br_4_-3,3′-Co(1,2-C_2_B_9_H_9_)_2_]^−^ anion has the *gauche* conformation stabilized by two pairs of the intramolecular CH···Br hydrogen bonds and two BH···Br contacts between the ligands (Figure 7). In turn, the presence of two signals of nonequivalent *CH* groups in the ^1^H NMR spectrum indicates the absence of free rotation of the dicarbollide ligands and the retention of the *gauche* configuration in solution [99].

The 4,4′,7,7′-tetraiodo derivative of cobalt bis(dicarbollide) [4,4′7,7′-I_4_-3,3′-Co(1,2-C_2_B_9_H_9_)_2_]^−^ was prepared in a similar way by the reaction of CoCl_2_ with [9,11-I_2_-*nido*-7,8-C_2_B_9_H_10_]^−^ in tetrahydrofuran and *t*-BuOK as a base. As in the case of the 4,4′,7,7′-tetrabromo derivative, the presence of two signals of nonequivalent *CH* groups in the ^1^H NMR spectrum indicates the absence of free rotation of the dicarbollide ligands and the retention of the *gauche* configuration in solution [90].

The use of asymmetrically substituted *nido*-carboranes leads to mixtures of the corresponding *rac*- and *meso*-isomers of transition metal bis(dicarbollides). Moreover, in the case of *rac*-isomers, due to asymmetric substitution in the dicarbollide ligand, as many as five rotational conformations of the bis(dicarbollide) anion differing in energy are possible. The mixture of 4,4′- and 4,7′-diiodo derivatives of cobalt bis(dicarbollide) *rac*-[4,4′-I_2_-3,3′-Co(1,2-C_2_B_9_H_10_)_2_]^−^ and *meso*-[4,7′-I_2_-3,3′-Co(1,2-C_2_B_9_H_10_)_2_]^−^ was prepared by the reaction of CoCl_2_ with [9-I-*nido*-7,8-C_2_B_9_H_11_]^−^ in 1,2-dimethoxyethane in the presence of *t*-BuOK as a base and separated by column chromatography on silica. Single-crystal X-ray diffraction study of Cs[4,4′-I_2_-3,3′-Co(1,2-C_2_B_9_H_10_)_2_] revealed that the *rac*-isomer adopts the so-called *gauche-2* conformation (the ligand rotation angle is 252°), stabilized by two pairs of the intramolecular CH···I hydrogen bonds (Figure 7) [88]. According to the quantum chemical calculations, the energy of this conformation is 6.4 and 7.4 kcal mol^–1^ lower than the energies of the *cisoid-2* (the ligand rotation angle is 324°) and *transoid* (the ligand rotation angle is 180°) conformations stabilized by two individual CH···I bonds, respectively, and by 11.9 and 12.0 kcal mol^–1^ below than the energies of the *cisoid-1* (the ligand rotation angle is 36°) and *gauche-1* (the ligand rotation angle is 108°) conformations, respectively, in which these interactions are completely absent. For the *meso*-isomer [4,7′-I_2_-3,3′-Co(1,2-C_2_B_9_H_10_)_2_]^−^, the most stable is the *gauche* conformation stabilized by one pair of the CH···I bonds [88].

**Figure 8:**
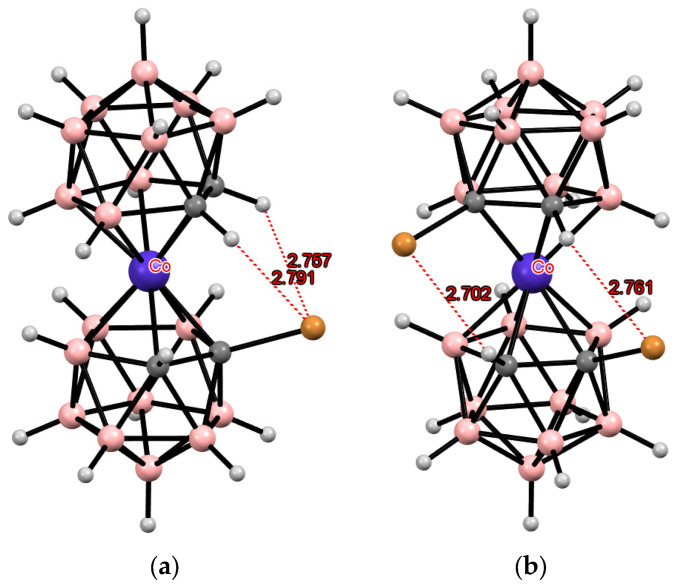
Intramolecular CH···Br hydrogen bonding in the solid-state structures of [1-Br-3,3′-Co(1,2-C_2_B_9_H_10_)(1′,2′-C_2_B_9_H_11_)]^−^ (**a**) and [1,1′-Br_2_-3,3′-Co(1,2-C_2_B_9_H_10_)_2_]^−^ (**b**) anions.

The *C*-substituted bromo derivatives [1-Br-3,3′-Co(1,2-C_2_B_9_H_10_)(1′,2′-C_2_B_9_H_11_)]^−^ and *rac*-[1,1′-Br_2_-3,3′-Co(1,2-C_2_B_9_H_10_)_2_]^−^ were prepared by the treatment of the *C*-lithium derivatives of cobalt bis(dicarbollide) with BrCH_2_CN and BrCN, respectively [100]. The solid-state structures of Cs[1-Br-3,3′-Co(1,2-C_2_B_9_H_10_)(1′,2′-C_2_B_9_H_11_)] and (Me_4_N)[1,1′-Br_2_-3,3′-Co(1,2-C_2_B_9_H_10_)_2_] were determined by single-crystal X-ray diffraction. In both compounds, the cobalt bis(dicarbollide) anions adopt the *cisoid* conformation. In the monobromo derivative, this conformation is stabilized by one pair of the intramolecular CH···Br hydrogen bonds, while in the dibromo derivative, the stabilization is achieved through two CH···Br bonds and two short BH···Br contacts (Figure 8) [100].

**Figure 9:**
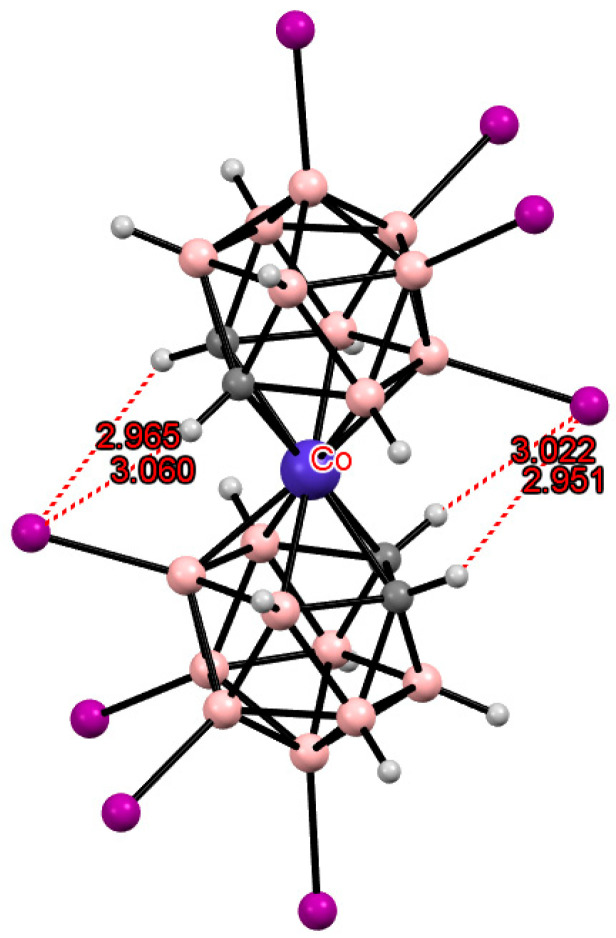
Intramolecular CH···I hydrogen bonding in the solid-state structure of the [8,8′,9,9′,10,10′,12,12′-I_8_-3,3′-Co(1,2-C_2_B_9_H_7_)_2_]^−^ anion.

An increase in the number of substituents due to their introduction into positions not involved in metal coordination does not lead to a change in the preference for one or another conformation. Thus, the 8,8′,9,9′,10,10′,12,12′-octaiodo derivative of cobalt bis(dicarbollide) [8,8′,9,9′,10,10′,12,12′-I_8_-3,3′-Co(1,2-C_2_B_9_H_7_)_2_]^−^ has the same *transoid* conformation as the 8,8′-diiodo derivative [8,8′-I_2_-3,3′-Co(1,2-C_2_B_9_H_10_)_2_]^−^ due to two pairs of the CH···I hydrogen bonds (Figure 9) [44]. In this case, a noticeable lengthening of the CH···I bonds is observed due to the increase in the metal–ligand distance caused by the weakening of the donor nature of the dicarbollide ligands upon the introduction of several electron-withdrawing substituents.

**Figure 10:**
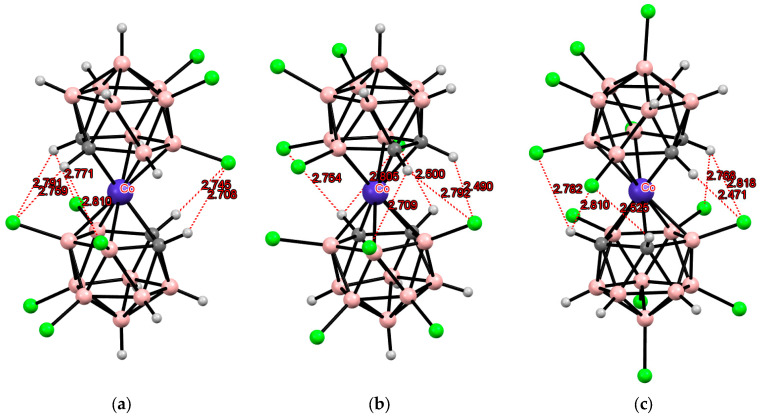
Intramolecular CH···Cl hydrogen bonding in the solid-state structures of [4,7,8,8′,9,9′,12,12′-Cl_8_-3,3′-Co(1,2-C_2_B_9_H_6_)(1′,2′-C_2_B_9_H_8_)]^−^ (**a**); [4,4′,7,7′,8,8′,9,9′,12,12′-Cl_10_-3,3′-Co(1,2-C_2_B_9_H_6_)_2_]^−^ (**b**); and [4,4′,7,7′,8,8′,9,9′,10,10′,12,12′-Cl_12_-3,3′-Co(1,2-C_2_B_9_H_5_)_2_]^−^ anions (**c**).

The structure of polychloro derivatives containing substituents at both position 8 and positions 4 and 7 of the dicarbollide ligand is of particular interest due to the possibility of the formation of both *transoid* and *gauche* conformers. A number of such derivatives were obtained by reacting cobalt bis(dicarbollide) with sulfuryl chloride in the presence of AlCl_3_. The solid-state structures of (Me_4_N)[4,7,8,8′,9,9′,12,12′-Cl_8_-3,3′-Co(1,2-C_2_B_9_H_6_)(1′,2′-C_2_B_9_H_8_)], (Me_4_N)[4,4′,7,7′,8,8′,9,9′,12,12′-Cl_10_-3,3′-Co(1,2-C_2_B_9_H_6_)_2_], and Cs[4,4′,7,7′,8,8′,9,9′,10,10′,12,12′-Cl_12_-3,3′-Co(1,2-C_2_B_9_H_5_)_2_] were determined by single-crystal X-ray diffraction [101]. The octachloro derivative [4,7,8,8′,9,9′,12,12′-Cl_8_-3,3′-Co(1,2-C_2_B_9_H_6_)(1′,2′-C_2_B_9_H_8_)]^−^ containing different amount of substituents in the C_2_B_3_ rings of the dicarbollide ligands adopts the *transoid* conformation stabilized by two pairs of CH···Cl hydrogen bonds, two single CH···Cl hydrogen bonds, and two short BH···Cl contacts (Figure 10). In contrast, the decachloro derivative [4,4′,7,7′,8,8′,9,9′,12,12′-Cl_10_-3,3′-Co(1,2-C_2_B_9_H_6_)_2_]^−^ adopts the *gauche* conformation stabilized by two pairs of CH···Cl hydrogen bonds and two single CH···Cl hydrogen bonds. The same conformation is observed in the case of the dodecachloro derivative [4,4′,7,7′,8,8′,9,9′,10,10′,12,12′-Cl_12_-3,3′-Co(1,2-C_2_B_9_H_5_)_2_]^−^ (Figure 10). It seems that it is precisely two additional BH···Cl contacts, absent in the second and third cases, that provide additional stabilization of the *transoid* conformation in the first case. ^1^H NMR spectroscopy data indicate that both the *transoid* and *gauche* conformations of these derivatives are retained in solution [101,102].

It is worth noting that in the solid state, under the influence of a crystalline environment, some 8-halogen and 8,8′-dihalogen derivatives of transition metal bis(dicarbollides) can take on a *gauche* or *cisoid* conformation. It was mentioned above that such a transformation occurs in the structure of (TMTTF)[8,8′-Cl_2_-3,3′-Co(1,2-C_2_B_9_H_10_)_2_]_2_ with rather weak intramolecular CH···Cl hydrogen bonds [87]. A similar picture is observed in some monobromo [8-O(CH_2_CH_2_)_2_S-8′-Br-3,3′-Co(1,2-C_2_B_9_H_10_)_2_] [103] and monoiodo Cs[8-HO-8′-I-3,3′-Co(1,2-C_2_B_9_H_10_)_2_]_2_ [104], [8-O(CH_2_CH_2_)_2_S-8′-I-3,3′-Co(1,2-C_2_B_9_H_10_)_2_] [105], [8-Et_3_PO-8′-I-3,3′-Co(1,2-C_2_B_9_H_10_)_2_] [106], [8-Ph_3_PO-8′-I-3,3′-Co(1,2-C_2_B_9_H_10_)_2_] [107], and [8-HC≡CCH_2_Me_2_N-8′-I-3,3′-Co(1,2-C_2_B_9_H_10_)_2_] [108] derivatives of cobalt bis(dicarbollide). At the same time, the ^1^H NMR spectra of the compounds indicate a *transoid* conformation (or free rotation of ligands) in solution.

## 4. Chalcogen Derivatives of Transition Metal Bis(Dicarbollides)

Another type of derivative that has been studied in detail recently is methylthio derivatives of transition metal bis(dicarbollides). To synthesize the methylthio derivatives, symmetric and asymmetric dimethylsulfonium derivatives of *nido*-carborane [10-Me_2_S-7,8-C_2_B_9_H_11_] and [9-Me_2_S-7,8-C_2_B_9_H_11_] were first prepared, which were then demethylated and used to construct the corresponding metallacarboranes, conversely,, as in the case of iron bis(dicarbollide) derivatives, metallacarboranes were first assembled and then demethylated. As a result, a complete set of isomers of *B*-substituted methylthio derivatives of cobalt, iron, and nickel bis(dicarbollides) was prepared.

**Figure 11:**
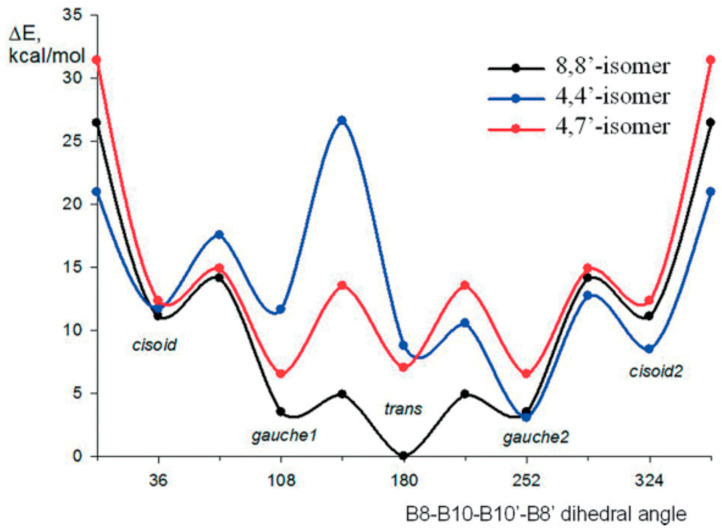
Energy profile for the rotation of dicarbollide ligands in the bis(methylthio) derivatives of cobalt bis(dicarbollide). Reproduced with permission from reference [109]. Copyright © (2017) John Wiley & Sons.

According to quantum chemical calculations, for the 8,8′-bis(methylthio) derivative of cobalt bis(dicarbollide) [8,8′-(MeS)_2_-3,3′-Co(1,2-C_2_B_9_H_11_)_2_]^−^, as well as for the 8,8′-diiodo derivative, the *transoid* conformation is the most preferred energetically. For the 4,4′-isomer [4,4′-(MeS)_2_-3,3′-Co(1,2-C_2_B_9_H_11_)_2_]^−^, the *gauche-1* conformation is somewhat more preferable, while for the 4,7′-isomer [4,7′-(MeS)_2_-3,3′-Co(1,2-C_2_B_9_H_11_)_2_]^−^, a pronounced energy minimum corresponds to the *gauche-2* conformation (Figure 11) [109].

**Figure 12:**
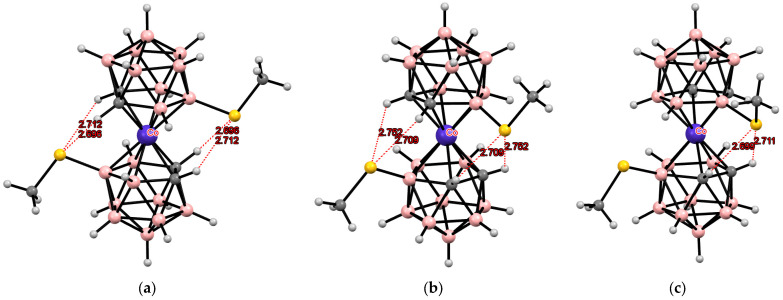
Intramolecular CH···S hydrogen bonding in the solid-state structures of [8,8′-(MeS)_2_-3,3′-Co(1,2-C_2_B_9_H_10_)_2_]^−^ (**a**); [4,4′-(MeS)_2_-3,3′-Co(1,2-C_2_B_9_H_10_)_2_]^−^ (**b**); and [4,7′-(MeS)_2_-3,3′-Co(1,2-C_2_B_9_H_10_)_2_]^−^ (**c**) anions.

Indeed, all these isomers have been synthesized, and their structures as tetrabutylammonium salts have been determined by single-crystal X-ray diffraction. The *transoid* conformation in the 8,8′-isomer and the *gauche-1* conformation in the 4,4′-isomer are stabilized by two pairs of the CH···S intramolecular hydrogen bonds, while the *gauche* conformation of the 4,7′-isomer is stabilized by one pair of the CH···S hydrogen bonds and one weak CH···S interaction (Figure 12) [109]. It is worth noting that according to NMR spectroscopy data, the *transoid* conformation of [8,8′-(MeS)_2_-3,3′-Co(1,2-C_2_B_9_H_11_)_2_]^−^ is retained in solution [98].

**Figure 13:**
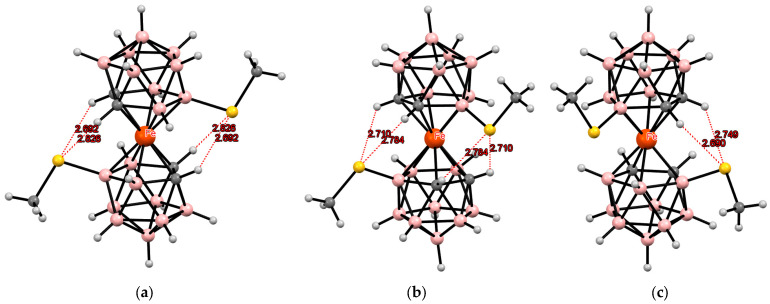
Intramolecular CH···S hydrogen bonding in the solid-state structures of [8,8′-(MeS)_2_-3,3′-Fe(1,2-C_2_B_9_H_10_)_2_]^2−^ (**a**); [4,4′-(MeS)_2_-3,3′-Fe(1,2-C_2_B_9_H_10_)_2_]^−^ (**b**); and [4,7′-(MeS)_2_-3,3′-Fe(1,2-C_2_B_9_H_10_)_2_]^−^ (**c**) anions.

**Figure 14:**
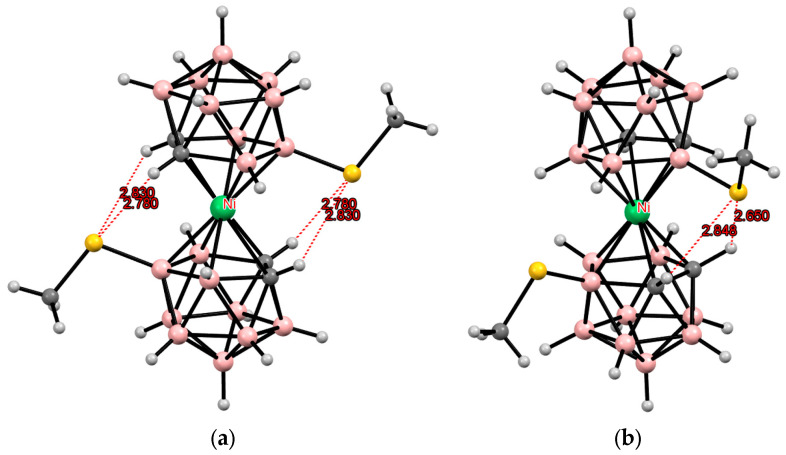
Intramolecular CH···S hydrogen bonding in the solid-state structures of [8,8′-(MeS)_2_-3,3′-Ni(1,2-C_2_B_9_H_10_)_2_]^−^ (**a**) and [4,7′-(MeS)_2_-3,3′-Ni(1,2-C_2_B_9_H_10_)_2_]^−^ (**b**) anions.

Similar structures were found in the tetrabutylammonium salts of the corresponding bis(methylthio) derivatives of iron and nickel bis(dicarbollides) (Figure 13 and Figure 14) [110,111].

**Figure 15:**
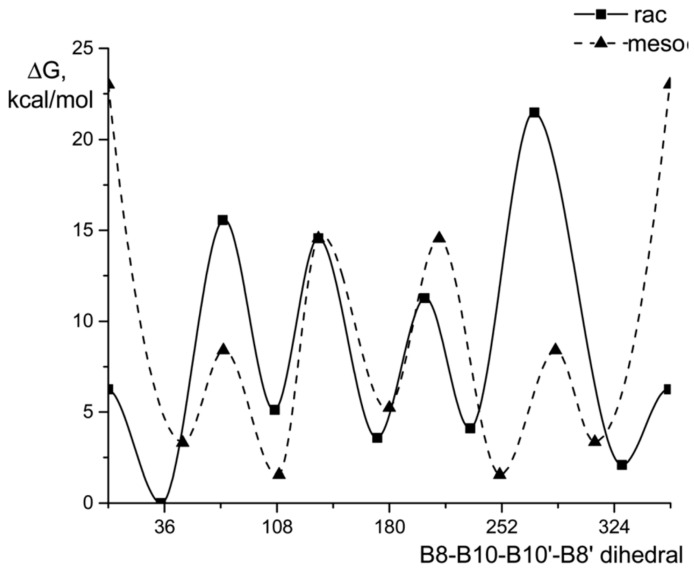
Energy profile for the rotation of dicarbollide ligands in 1,1′- and 1,2′-bis(methylthio) derivatives of cobalt bis(dicarbollide). Reproduced from reference [112] with permission of The Royal Society of Chemistry.

In the case of *C*-substituted methylthio derivatives of cobalt bis(dicarbollide), according to quantum chemical calculations, the *cisoid-1* conformation is the most energetically favorable for the 1,1′-isomer, while the *gauche* conformation is more preferable for the 1,2′-isomer (Figure 15) [112].

**Figure 16:**
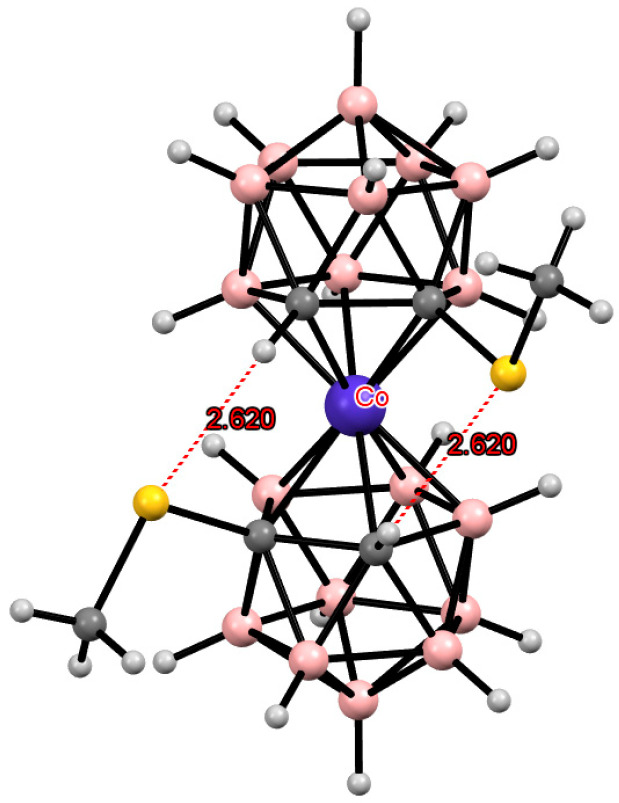
Intramolecular CH···S hydrogen bonding in the solid-state structure of the [1,1′-(MeS)_2_-3,3′-Co(1,2-C_2_B_9_H_10_)_2_]^−^ anion.

Indeed, the *cisoid-1* conformation stabilized by two hydrogen CH···S bonds and two short BH···S(Me) contacts was found in the crystal structure of (BEDT-TTF)[1,1′-(MeS)_2_-3,3′-Co(1,2-C_2_B_9_H_10_)_2_] (Figure 16) [112].

**Figure 17:**
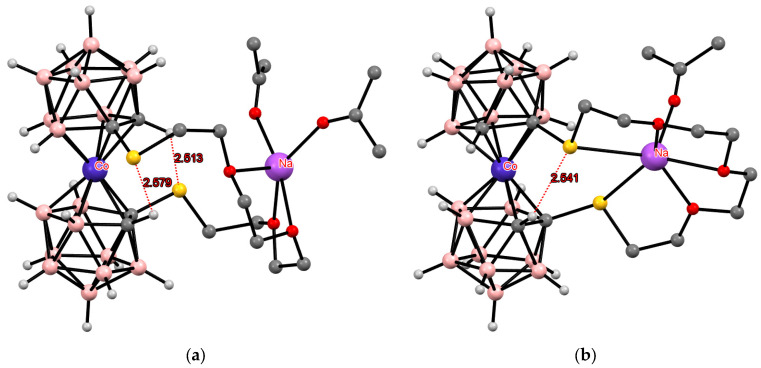
The solid-state structures of {(Me_2_CO)_2_Na[1,1′-μ-O(CH_2_CH_2_OCH_2_CH_2_S)_2_-3,3′-Co(1,2-C_2_B_9_H_10_)_2_]} (**a**) and {(Me_2_CO)Na[1,2′-μ-O(CH_2_CH_2_OCH_2_CH_2_S)_2_-3,3′-Co(1,2-C_2_B_9_H_10_)_2_]} (**b**). Hydrogen atoms of organic substituents are omitted for clarity.

Somewhat unexpectedly, the same conformation with two intramolecular CH···S hydrogen bonds was found in the structure of thia-crown ether with embedded cobalt bis(dicarbollide) fragment {(Me_2_CO)_2_Na[1,1′-μ-O(CH_2_CH_2_OCH_2_CH_2_S)_2_-3,3′-Co(1,2-C_2_B_9_H_10_)_2_]}. It is interesting that in the *meso*-isomer {(Me_2_CO)Na[1,2′-μ-O(CH_2_CH_2_OCH_2_CH_2_S)_2_-3,3′-Co(1,2-C_2_B_9_H_10_)_2_]}, in which the sulfur atoms are involved in the coordination of the sodium cation, one of them is also involved in the formation of the intramolecular CH···S hydrogen bond (Figure 17) [113]. However, it must be remembered that in this case, the geometry of the anion is determined mainly by the coordination of the sodium atom by the thia-crown.

It should be noted that in the solid state, under the influence of the crystalline environment, bis(alkylthio) derivatives of cobalt bis(dicarbollide) [8-RS-8′-R’S-3,3′-Co(1,2-C_2_B_9_H_10_)_2_]^−^ containing large substituents can take on other conformations, in particular the *gauche* conformation, an example of which is [8-(1″-EtOOCCH_2_-3″-Me-1″,3″-N_2_C_3_H_2_-2″-)CH_2_S-8′-EtS-3,3′-(1,2-C_2_B_9_H_10_)_2_] [114]. At the same time, the ^1^H NMR spectra of the compounds indicate a *transoid* conformation (or free rotation of ligands) in solution.

**Figure 18:**
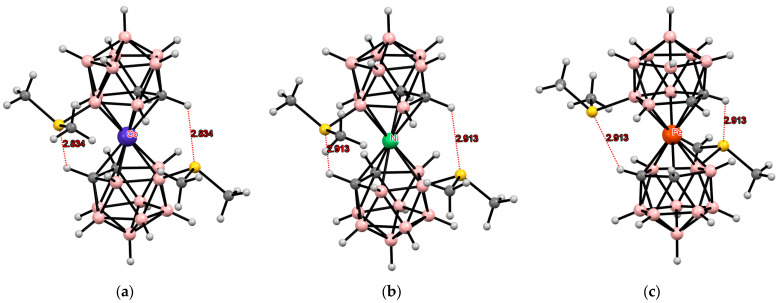
Intramolecular CH···S hydrogen bonding in the solid-state structures of [8,8′-(Me_2_S)_2_-3,3′-Co(1,2-C_2_B_9_H_10_)_2_] (**a**); [8,8′-(Me_2_S)_2_-3,3′-Ni(1,2-C_2_B_9_H_10_)_2_] (**b**); and [8,8′-(Me_2_S)_2_-3,3′-Fe(1,2-C_2_B_9_H_10_)_2_] (**c**).

**Figure 19:**
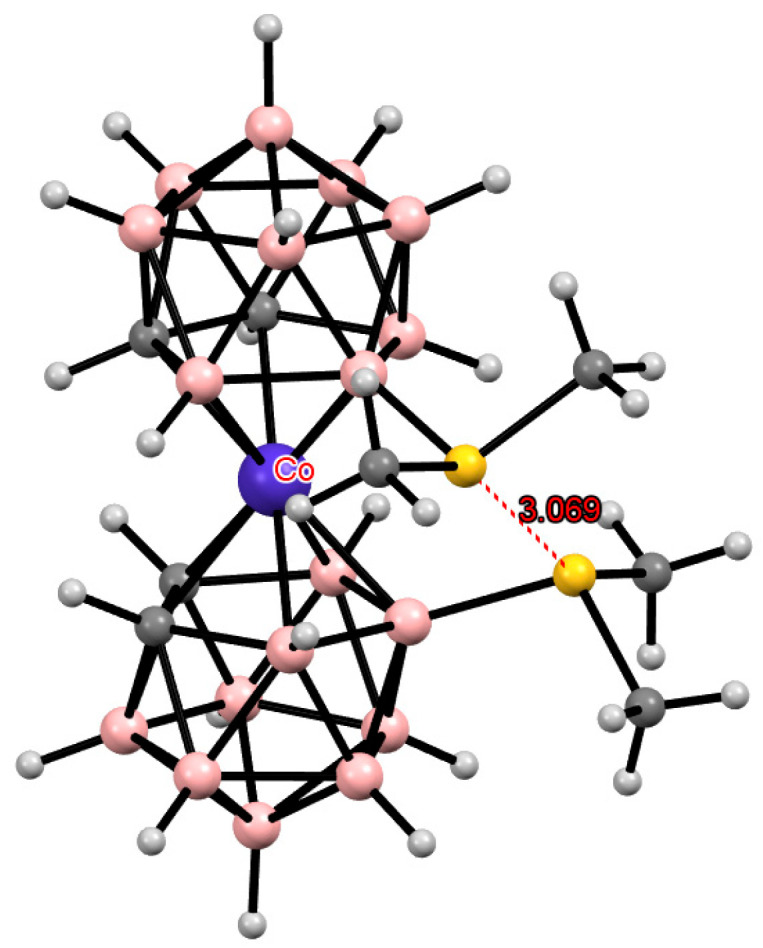
The solid-state structure of [8,8′-(Me_2_S)_2_-3,3′-Co(1,2-C_2_B_9_H_10_)_2_]Cl.

Each sulfur atom in the bis(methylthio) derivatives has two lone pairs of electrons and is, therefore, capable of forming intramolecular hydrogen bonds with two CH groups of the opposite dicarbollide ligand. In the case of bis(dimethylsulfonium) derivatives, each sulfur atom has only one lone pair of electrons and, therefore, can form only one hydrogen bond with the CH group of the opposite ligand. According to quantum chemical calculations, the gauche conformation is most preferred for the 8,8′-bis(dimethylsulfonium) derivative of cobalt(II) bis(dicarbollide) [115]. It is precisely this conformation, stabilized by two intramolecular CH···S hydrogen bonds, that was found in the crystal structure of [8,8′-(Me_2_S)_2_-3,3′-Co(1,2-C_2_B_9_H_11_)_2_] (Figure 18) [115]. However, for the similar derivative of cobalt(III) bis(dicarbollide), the cisoid conformation is energetically more favorable. It is this, stabilized by intramolecular S···S interactions, was found in the crystal structure of [8,8′-(Me_2_S)_2_-3,3′-Co(1,2-C_2_B_9_H_11_)_2_]Cl (Figure 19) [115].

According to quantum chemical calculations, for the 8,8′-bis(dimethylsulfonium) derivative of nickel(II) bis(dicarbollide) as well as for the similar derivative of cobalt(II) bis(dicarbollide), the most energetically favorable conformation is the gauche conformation. Indeed, this conformation, stabilized by two intramolecular CH···S hydrogen bonds, was found in the crystal structure of [8,8′-(Me_2_S)_2_-3,3′-Ni(1,2-C_2_B_9_H_11_)_2_] (Figure 18) [115].

The same gauche conformation, stabilized by two intramolecular hydrogen bonds CH···S, was also found in the crystal structure of the 8,8′-bis(dimethylsulfonium) derivative of iron(II) bis(dicarbollide) [8,8′-(Me_2_S)_2_-3,3′-Fe(1,2-C_2_B_9_H_11_)_2_] (Figure 18) [116].

**Figure 20:**
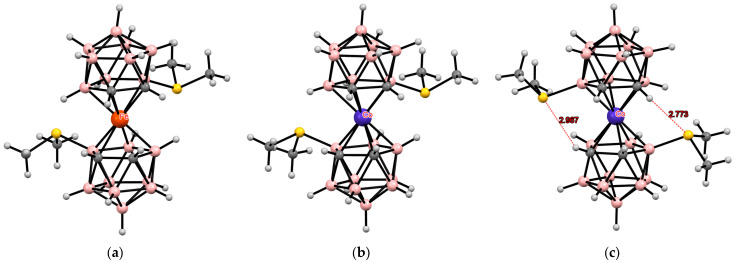
The solid-state structures of [4,4′-(Me_2_S)_2_-3,3′-Fe(1,2-C_2_B_9_H_10_)_2_] (*cisoid-1* conformation) (**a**); [4,4′-(Me_2_S)_2_-3,3′-Co(1,2-C_2_B_9_H_10_)_2_]^+^ (*cisoid-1* conformation) (**b**); and [4,4′-(Me_2_S)_2_-3,3′-Co(1,2-C_2_B_9_H_10_)_2_]^+^ (*cisoid-2* conformation) (**c**).

A more complex picture is observed in asymmetrically substituted dimethylsulfonium derivatives of bis(dicarbollide) complexes. In the case of iron(II) bis(dicarbollide), the 4,4′-isomer [4,4′-(Me_2_S)_2_-3,3′-Fe(1,2-C_2_B_9_H_11_)_2_] in the crystal adopts the cisoid-1 conformation, in which there are no significant interligand interactions (Figure 20) [117]. At the same time, in the structure of the isoelectronic cobalt(III) complex [4,4′-(Me_2_S)_2_-3,3′-Co(1,2-C_2_B_9_H_11_)_2_](BF_4_), two independent bis(dicarbollide) anions were found in the unit cell, one of which has the similar cisoid-1 conformation, whereas the second adopts the cisoid-2 conformation stabilized by two intramolecular CH···S hydrogen bonds (Figure 20) [118].

**Figure 21:**
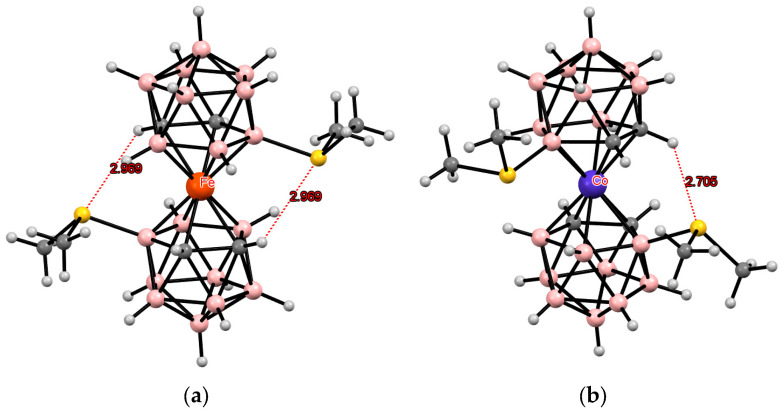
Intramolecular CH···S hydrogen bonding in the solid-state structures of [4,7′-(Me_2_S)_2_-3,3′-Fe(1,2-C_2_B_9_H_10_)_2_] (**a**) and [4,7′-(Me_2_S)_2_-3,3′-Co(1,2-C_2_B_9_H_10_)_2_]^−^ (**b**).

Again, in the case of iron(II) bis(dicarbollide), the 4,7′-isomer [4,7′-(Me_2_S)_2_-3,3′-Fe(1,2-C_2_B_9_H_11_)_2_] adopts the transoid conformation stabilized by two intramolecular CH···S hydrogen bonds (Figure 21) [119], whereas the 4,7′-isomer of the isoelectronic cobalt(II) bis(dicarbollide) in the crystal of [4,7′-(Me_2_S)_2_-3,3′-Co(1,2-C_2_B_9_H_11_)_2_](BF_4_) has the gauche conformation stabilized by one intramolecular CH···S hydrogenhydrogen bond and one BH···S contact (Figure 21) [118]. Such conformational differences in isoelectronic complexes indicate the very weak nature of intramolecular interactions between dicarbollide ligands in them and a large role of the crystalline environment (presence or absence of counterions).

Not surprisingly, no significant interactions between dicarbollide ligands were detected in the cobalt bis(dicarbollide) derivative containing a single dimethylsulfonium substituent [4-Me_2_S-3,3′-Co(1,2-C_2_B_9_H_10_)(1′,2′-C_2_B_9_H_11_)] [118].

As in the case of halogen derivatives, the introduction of additional substituents into the dicarbollide ligand belt distant from the metal atom does not have a noticeable effect on the conformation of bis(dicarbollide) complexes. For example, in the solid state, [6,6′-Ph_2_-8,8′-(Me_2_S)_2_-3,3′-Fe(1,2-C_2_B_9_H_10_)_2_] has the *transoid* conformation [120], and [12,12′-(ClHg)_2_-4,4′-(Me_2_S)_2_-3,3′-Fe(1,2-C_2_B_9_H_10_)_2_] has the *cisoid* conformation [121], which are characteristic of the bis(dimethylsulfonium) derivatives that do not contain additional substituents.

**Figure 22:**
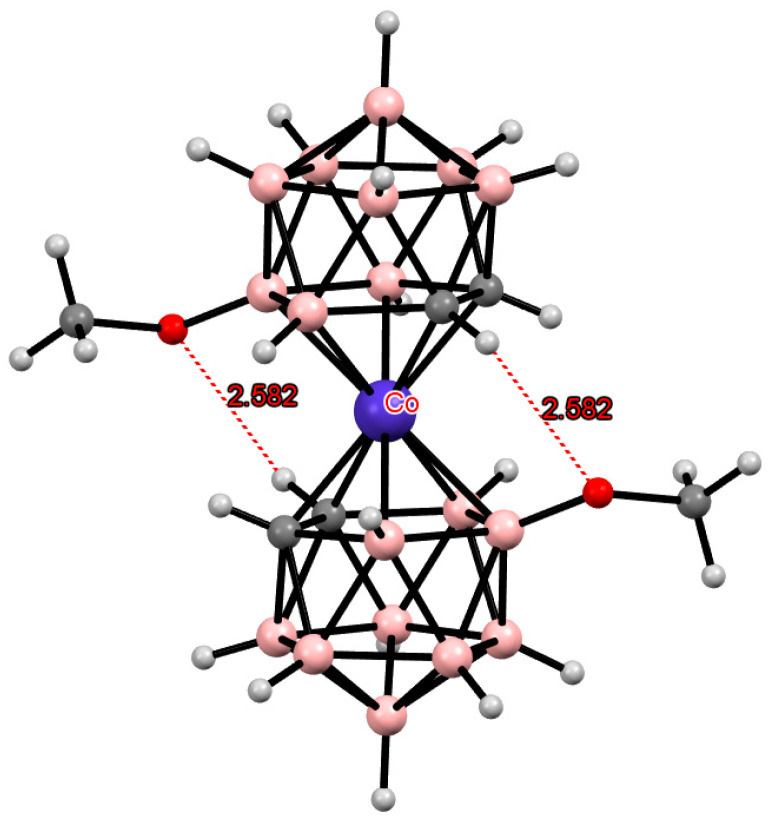
Intramolecular CH···O hydrogen bonding in the solid-state structure of the [8,8′-(MeO)_2_-3,3′-Co(1,2-C_2_B_9_H_10_)_2_]^−^ anion.

One would expect that in the 8,8′-di(methoxy) derivative of cobalt bis(dicarbollide), as in the 8,8′-difluoro derivative, there would be no interactions between the substituents in the opposite dicarbollide ligands. However, in the crystal structure of (Me_4_N)[8,8′-(MeO)_2_-3,3′-Co(1,2-C_2_B_9_H_11_)_2_], the transoid conformation of the anion was found to be stabilized by two intramolecular CH···O hydrogen bonds (Figure 22). This difference may apparently be caused by the larger covalent radius of oxygen compared with fluorine. In contrast to the similar bis(methylsulfide) derivative where both lone pairs of the sulfur atom are oriented toward the corresponding hydrogen atoms (Figure 12a), in the di(methoxy) derivative, only one lone pair of the oxygen atom is directed toward the CH group, and the other looks away. This orientation is expressed in a well-defined rotation of the methoxy group (angle of about 70°) [122]. As expected, according to ^1^H NMR spectroscopy data, intramolecular hydrogen bonds in the 8,8′-di(methoxy) derivative of bis(cobalt dicarbollide) in solution are very weak and significantly weaker than the hydrogen bonds in the 8,8′-dichloro derivative [98].

**Figure 23:**
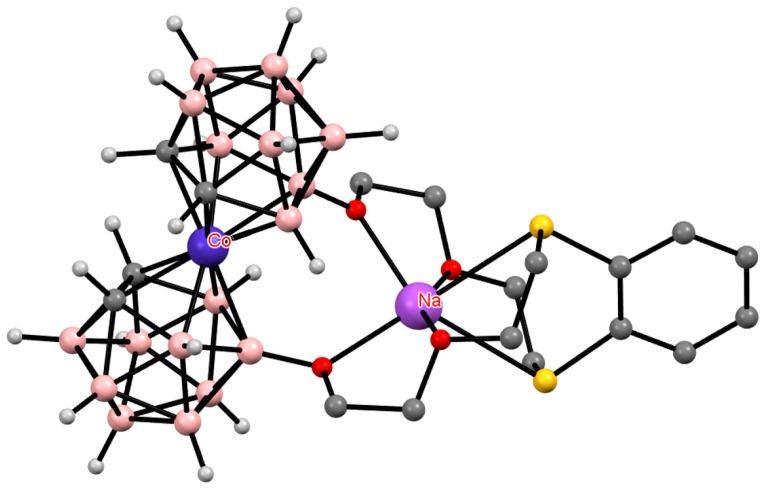
The solid-state structure of {Na[8,8′-μ-1″,2″-C_6_H_4_(SCH_2_CH_2_OCH_2_CH_2_O)_2_-3,3′-Co(1,2-C_2_B_9_H_10_)_2_]}. Hydrogen atoms of organic substituents are omitted for clarity.

Therefore, it is not surprising that the conformation of cobalt bis(dicarbollide) in structure of the thia-crown ether with an embedded cobalt bis(dicarbollide) fragment {Na[8,8′-μ-1″,2″-C_6_H_4_(SCH_2_CH_2_OCH_2_CH_2_O)_2_-3,3′-Co(1,2-C_2_B_9_H_10_)_2_]} is determined solely by the coordination of the sodium atom by the thia-crown (Figure 23) [123].

**Figure 24:**
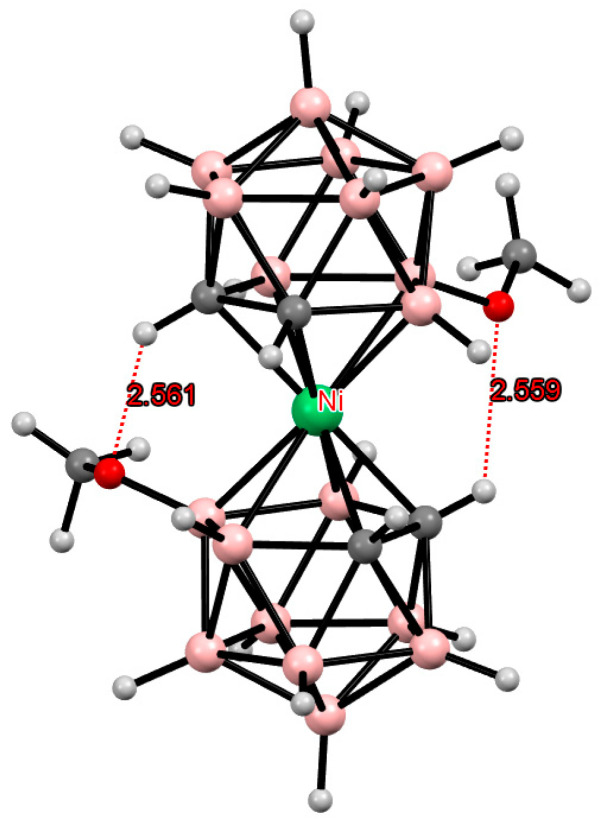
Intramolecular CH···O hydrogen bonding in the solid-state structure of [8,8′-(MeO)_2_-3,3′-Ni(1,2-C_2_B_9_H_10_)_2_].

In contrast to the 8,8′-di(methoxy) derivative of cobalt(III) bis(dicarbollide), the 8,8-di(methoxy) derivative of nickel(IV) bis(dicarbollide) )[8,8′-(MeO)_2_-3,3′-Ni(1,2-C_2_B_9_H_11_)_2_] adopts the gauche conformation, which is also stabilized by two intramolecular CH···O hydrogen bonds (Figure 24) [124].

It is obvious that the introduction of only one alkoxyor hydroxy substituent into the bis(dicarbollide) complex is not enough to effectively stabilize any conformation. Moreover, in the case of the tetramethylthiafulvalenium salt of the 8-hydroxy derivative of cobalt bis(dicarbollide) (TMTTF)[8-HO-3,3′-Co(1,2-C_2_B_9_H_10_)(1′,2′-C_2_B_9_H_11_)], the formation of not intramolecular but intermolecular CH···O hydrogen bonds between the anions is observed in the crystal [125].

However, if the second dicarbollide ligand contains substituents with acidic hydrogens, the formation of hydrogen bonds between the substituents is possible. This is precisely the picture observed in the structures of [8-MeO-8′-C_6_H_5_CH_2_NH_2_CH_2_-3,3′-Co(1,2-C_2_B_9_H_10_)_2_] [126], [8-MeO-8′-HOCH_2_CH_2_NH_2_CH_2_-3,3′-Co(1,2-C_2_B_9_H_10_)_2_] [127], [8-MeO-8′-C_5_H_5_NCH_2_-3,3′-Co(1,2-C_2_B_9_H_10_)_2_] [127], and [8-MeO-8′-Ph_3_PCH_2_-3,3′-Co(1,2-C_2_B_9_H_10_)_2_] [127] where the cisoid conformation is stabilized due to the formation of intramolecular NH···O or CH···O hydrogen bond with the participation of the methoxy group. However, it is unknown whether these intramolecular hydrogen bonds exist in solution.

**Figure 25:**
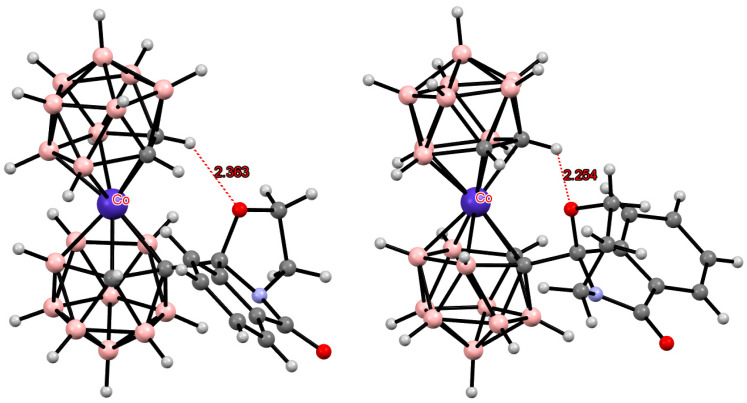
Intramolecular CH···O hydrogen bonding in cobalt bis(dicarbollide) derivatives.

The formation of intramolecular hydrogen bonds is also possible when a short spacer appears between the dicarbollide ligand and the oxygen atom. This allows the latter to move closer to the CH groups of another dicarbollide ligand. Indeed, strong intramolecular CH···O hydrogen bonds have been found in crystal structures of cobalt bis(dicarbollide) derivatives with sterically rigid substituents, in which there is a monoatomic carbon brigge between the carbon atom of the dicarbollide ligand and the oxygen atom (Figure 25) [128]. However, in the case of flexible substituents such as HOCH_2_- and HOCH_2_CH_2_-, the formation of intermolecular rather than intramolecular hydrogen bonds is more preferable [129].

It is not surprising that the oxygen atoms in 8- and 8,8′-substituted cyclic oxonium derivatives bis(dicarbollide) complexes of cobalt [130,131] and iron [45,132] do not participate in the formation of intramolecular hydrogen bonds.

## 5. Pnictogen Derivatives of Transition Metal Bis(Dicarbollides)

**Figure 26:**
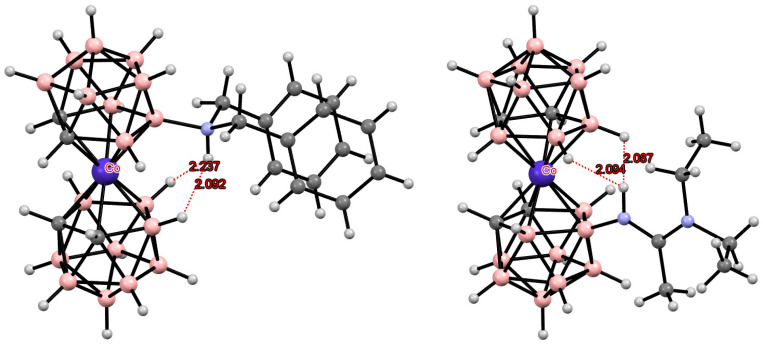
Intramolecular NH···HB dihydrogen bonding in cobalt bis(dicarbollide) derivatives.

The electron-donating effect of the metallacarborane cluster leads to easy protonation of the amino group directly bonded to the boron atom [133]. Therefore, the hydrogen atom in the amino derivatives of bis(dicarbollide) transition metal complexes cannot act as an acceptor of a hydrogen bond with the CH groups of another dicarbollide ligand. However, the acidic NH hydrogen is capable of forming intramolecular N-H...H-B dihydrogen bonds, thus stabilizing certain conformations of bis(dicarbollide) complexes. Such interactions were found in the crystal structures of the dibenzylamino derivative [8-(C_6_H_5_CH_2_)_2_NH-3,3′-Co(1,2-C_2_B_9_H_10_)(1′,2′-C_2_B_9_H_11_)] [133] and amidines [8-R’_2_NC(R)NH-3,3′-Co(1,2-C_2_B_9_H_10_)(1′,2′-C_2_B_9_H_11_)] (R = Me, R’ = Et; R = Et, R’ = Me, Et; R = Et, R’_2_ = (CH_2_)_5_) (Figure 26) [133,134].

**Figure 27:**
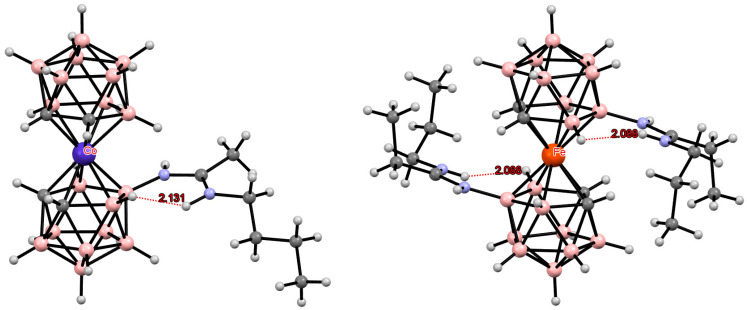
Intramolecular NH···HB dihydrogen bonding in amidine derivatives of transition metal bis(dicarbollide) complexes.

However, in the case of amidines with two NH groups, such as [8-BuHNC(Me)NH-3,3′-Co(1,2-C_2_B_9_H_10_)(1′,2′-C_2_B_9_H_11_)] [133] and [8,8′-(PrHNC(Et)NH)_2_-3,3′-Fe(1,2-C_2_B_9_H_10_)_2_] [45], the formation of intramolecular N-H...H-B dihydrogen bonds with the BH groups of the same dicarbollide ligand is more preferable (Figure 27). In any case, these dihydrogen bonds are rather weak and unlikely to exist in solution.

**Figure 28:**
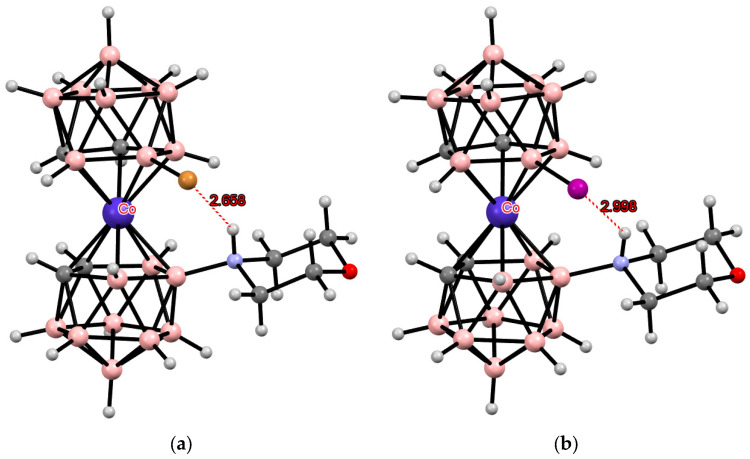
Intramolecular NH···X hydrogen bonding in [8-O(CH_2_CH_2_)_2_NH-8′-Br-3,3′-Co(1,2-C_2_B_9_H_10_)_2_] (**a**) and [8-O(CH_2_CH_2_)_2_NH-8′-I-3,3′-Co(1,2-C_2_B_9_H_10_)_2_] (**b**).

Another example of the formation of the intramolecular hydrogen bond with the participation of acidic NH hydrogen in ammonium derivatives of cobalt bis(dicarbollide) is the solid-state structure of [8-O(CH_2_CH_2_)_2_NH-8′-X-3,3′-Co(1,2-C_2_B_9_H_10_)_2_] (X = Br, I) where the cisoid conformation is stabilized by the intramolecular NH...X hydrogen bond (Figure 28) [103,107].

**Figure 29:**
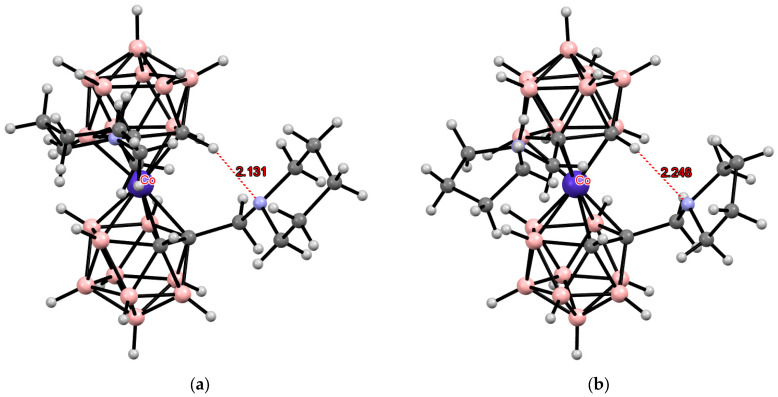
Intramolecular CH···N hydrogen bonding in piperidine (**a**) and pyrollidine (**b**) derivatives of cobalt bis(dicarbollide).

The formation of intramolecular hydrogen bonds is possible when a short spacer appears between the dicarbollide ligand and the nitrogen atom. This allows the latter to approach the CH groups of another dicarbollide ligand. Such intramolecular CH···N hydrogen bonds have been found in the solid-state structures of cobalt bis(dicarbollide) derivatives containing pyrollidine or piperidine substituents [1-(CH_2_)_n_NHCH_2_-1′- (CH_2_)_n_NCH_2_-3,3′-Co(1,2-C_2_B_9_H_10_)_2_] (n = 4, 5) (Figure 29). Each of the dicarbollide ligands contains a substituent in which the nitrogen atom is linked to the carbon atom of the carborane cage through a methylene spacer. The nitrogen atom in one of the substituents is protonated, and in the second, it participates in the formation of the intramolecular CH···N hydrogen bond with the CH group of the second dicarbollide ligand [135].

**Figure 30:**
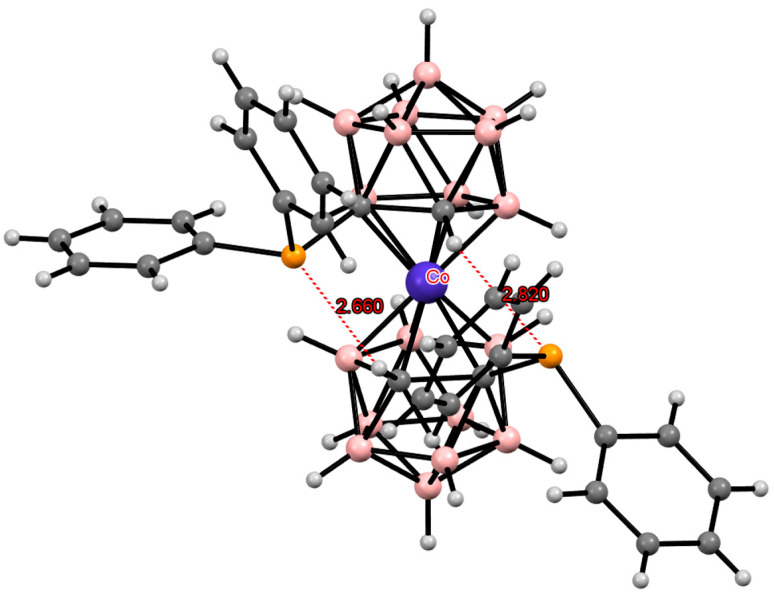
Intramolecular CH···P hydrogen bonding in the solid-state structure of the [1,1′-(Ph_2_P)_2_-3,3′-Co(1,2-C_2_B_9_H_10_)_2_]^−^ anion.

The intramolecular CH...P hydrogen bonds are found in the structure of (Me_4_N)[1,1′-(Ph_2_P)_2_-3,3′-Co(1,2-C_2_B_9_H_10_)_2_] where two such bonds stabilize the *cisoid-1* conformation of the bis(dicarbollide) anion (Figure 30) [136].

## 6. Aryl Derivatives of Transition Metal Bis(Dicarbollides)

**Figure 31:**
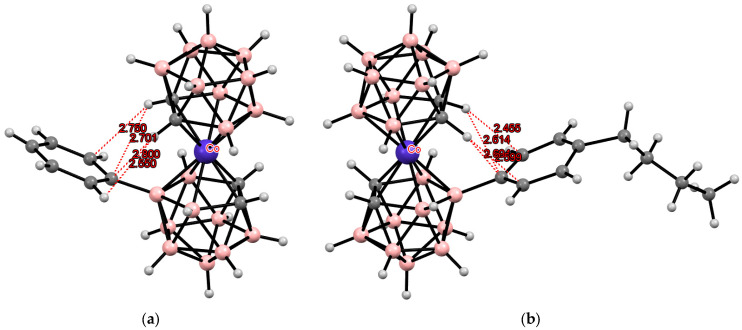
Intramolecular CH···π hydrogen bonding in [8-Ph-3,3′-Co(1,2-C_2_B_9_H_10_)(1′,2′-C_2_B_9_H_11_)]^−^ (**a**) and [8-(4′’-BuC_6_H_4_)-3,3′-Co(1,2-C_2_B_9_H_10_)(1′,2′-C_2_B_9_H_11_)]^−^ (**b**) anions.

Another type of rotamer stabilization was found in the structures of aryl derivatives of cobalt bis(dicarbollide) [8-Ar-3,3′-Co(1,2-C_2_B_9_H_10_)(1′,2′-C_2_B_9_H_11_)]^−^ (Ar = C_6_H_5_, C_6_H_4_-4-Bu) where stabilization of the *transoid* conformation is achieved through the formation of intramolecular CH···π bonds between acidic CH groups in one dicarbollide ligand and phenyl substituents in the other one (Figure 31) [130,137].

**Figure 32:**
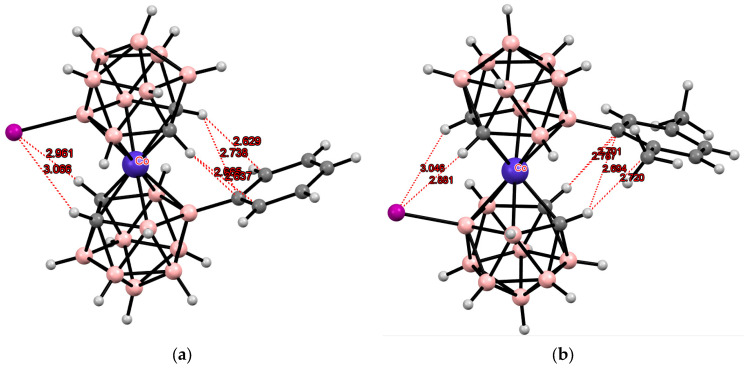
Intramolecular hydrogen bonding in [8-C_6_H_5_-8′-I-3,3′-Co(1,2-C_2_B_9_H_10_)_2_]^−^ (**a**) and [8-(2″,5″-Me_2_C_6_H_3_)-8′-I-3,3′-Co(1,2-C_2_B_9_H_10_)_2_]^−^ (**b**) anions.

Some later derivatives with the “double-locked” *transoid* conformation due to the intramolecular CH···I and CH···π hydrogen bonds between the dicarbollide ligands [8-Ar-8′-I-3,3′-Co(1,2-C_2_B_9_H_10_)_2_]^−^ (Ar = C_6_H_5_ [107], C_6_H_3_-2,5-Me_2_ [106], C_6_H_3_-3,4-Cl_2_ [106]) were described (Figure 32). It was demonstrated that the intramolecular CH···π hydrogen bonds exist not only in the solid state but in solution as well [106].

**Figure 33:**
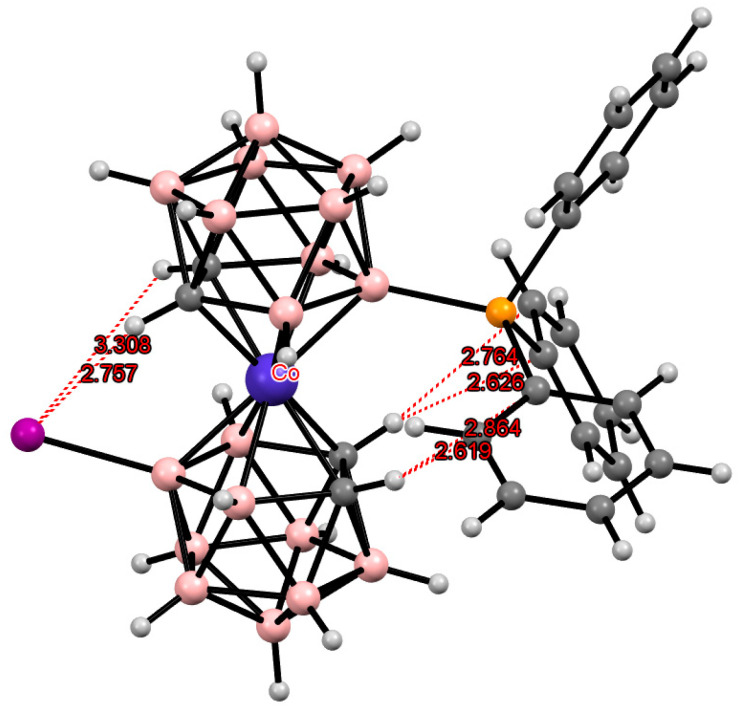
Intramolecular hydrogen bonding in the solid-state structure of [8-Ph_3_P-8′-I-3,3′-Co(1,2-C_2_B_9_H_10_)_2_].

Another example of the “double-locked” transoid conformation due to the intramolecular CH···I and CH···π hydrogen bonds was found in the crystal structure of [8-Ph_3_P-8′-I-3,3′-Co(1,2-C_2_B_9_H_10_)_2_] (Figure 33) [107].

## 7. BH···M Interactions in Derivatives of Transition Metal Bis(Dicarbollides)

**Figure 34:**
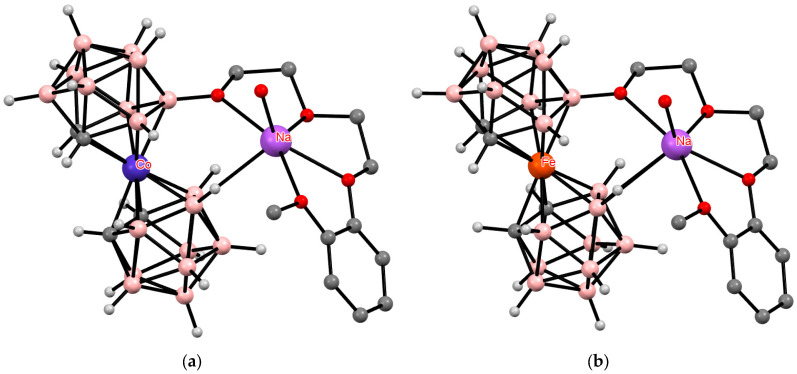
The solid-state structures of {(H_2_O)Na[8-(2″-MeOC_6_H_4_O(CH_2_CH_2_O)_2_)-3,3′-Co(1,2-C_2_B_9_H_10_)(1′,2′-C_2_B_9_H_11_)]} (**a**) and {(H_2_O)Na[8-(2″-MeOC_6_H_4_O(CH_2_CH_2_O)_2_)-3,3′-Fe(1,2-C_2_B_9_H_10_)(1′,2′-C_2_B_9_H_11_)]} (**b**). Hydrogen atoms of organic substituents and water are omitted for clarity.

An interesting example of stabilization of the cisoid conformation is the coordination of alkali metal cations by the open dioxane ring in the solid-state structures of the complexes [8-R(OCH_2_CH_2_)_2_O-3,3′-M(1,2-C_2_B_9_H_10_)(1′,2′-C_2_B_9_H_11_)]^−^ (M = Co, R = Et, Ac, C_6_H_4_-2-OMe; M = Fe, R = C_6_H_4_-2-OMe) [113,132,138,139]. In this case, the deficiency of oxygen atoms in the metal coordination sphere is compensated by the coordination of the most hydride BH groups of the unsubstituted dicarbollide ligand, resulting in stabilization of the *cisoid* conformation (Figure 34). A similar picture is observed in compounds containing two [140], three [139], and four [141] cobalt bis(dicarbollide) fragments.

It should be noted that in the absence of alkali metal cations, the bis(dicarbollide) fragment adopts a predominantly *transoid* conformation, which is characteristic of intramolecular salts [8-L(CH_2_CH_2_O)_2_-3,3′-M(1,2-C_2_B_9_H_10_)(1′,2′-C_2_B_9_H_11_)] (M = Co, L = Et_3_N, Py; M = Fe, L = Ph_3_P, Py) [132,142].

## 8. Interligand Interactions and Properties of Bis(Dicarbollide) Complexes

The effect of interligand interactions in transition metal bis(dicarbollide) complexes on their properties should be considered in two different aspects. The first is static and is associated with the difference in dipole moments of different rotamers. The dipole moment of bis(dicarbollide) anions has a direct effect on the crystalline packing of tetrathiafulvalene salts based on them, which, in turn, determines their electrical conductive properties [143,144].

**Figure 35:**
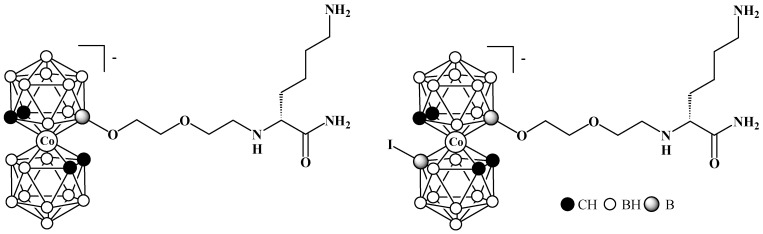
Lysine derivatives of cobalt bis(dicarbollide).

Much less obvious is the effect of the dipole moment of the cobalt bis(dicarbollide) complex on the biological properties of its derivatives. It would seem that the introduction of an iodine atom into cobalt bis(dicarbollide), located far from the biologically active part of the molecule, should not have a noticeable effect on its biological properties. All the more surprising is the 8-fold increase in the antibacterial activity of the iodinated lysine derivative of cobalt bis(dicarbollide) (Figure 35) against Staphylococcus aureus compared with the non-iodinated analog (MBC = 0.98 and 7.81 μM, respectively). It is obvious that this increase in antibacterial activity correlates well with the increase in lipophilicity of the iodinated compound (the octanol−water partition coefficient log P are 3.02 and 1.94, respectively) [145]. Moreover, it was found that iodination of cobalt bis(dicarbollide) itself also leads to a significant increase in its lipophilicity (log P are 1.32 and 2.68, for the parent cobalt bis(dicarbollide) and the 8-iodo derivative) [145]. We believe that this increase in lipophilicity is due to the formation of the stable *transoid* conformation, which has a lower dipole moment than the parent anion. To date, a number of iodinated cobalt bis(dicarbollide) derivatives have been obtained, many of which exhibit high antibacterial activity [145,146,147,148,149]. However, to better understand the effect of iodination on their biological behavior, a more detailed comparison of their properties with those of non-iodinated compounds is required.

**Figure 36:**
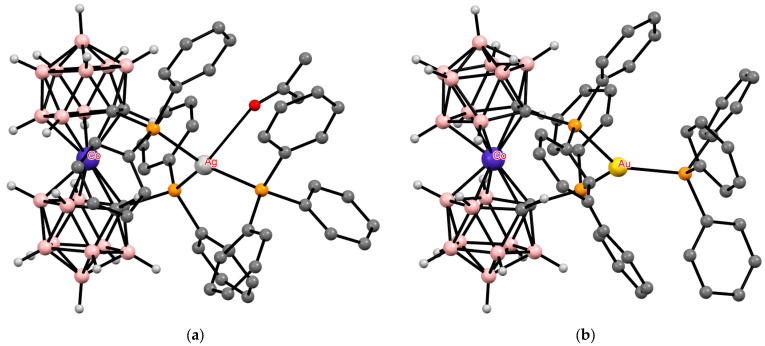
The solid-state structures of {(Me_2_CO)(Ph_3_P)Ag[1,1′-(Ph_2_P)_2_-3,3′-Co(1,2-C_2_B_9_H_10_)_2_]} (**a**) and {(Ph_3_P)Au[1,1′-(Ph_2_P)_2_-3,3′-Co(1,2-C_2_B_9_H_10_)_2_]} (**b**). Hydrogen atoms of organic ligands are omitted for clarity.

The other aspect is dynamic: intramolecular hydrogen bonds are rather weak and easily broken by an external impact. Such an impact can be the presence of external transition metal ions or complexes. In the presence of external complexing metals, weak intramolecular L···HC hydrogen bonds will be broken to form stronger L—>M donor–acceptor bonds with the metal ion. An example of such a process is the complexation of the 1,1′-bis(diphenylphosphine) derivative of cobalt bis(dicarbollide) [1,1′-(Ph_2_P)_2_-3,3′-Co(1,2-C_2_B_9_H_10_)_2_]^−^ with silver and gold complexes [(Ph_3_P)MCl] (M = Ag, Au), leading to the rupture of intramolecular CH···P hydrogen bonds and the formation of P—>M donor–acceptor bonds (Figure 36). However, the complexation leads only to a slight rotation of dicarbollide ligands compared with the starting phosphine (24.7° and 47.9° for silver and gold complexes, respectively) [136].

**Figure 37:**
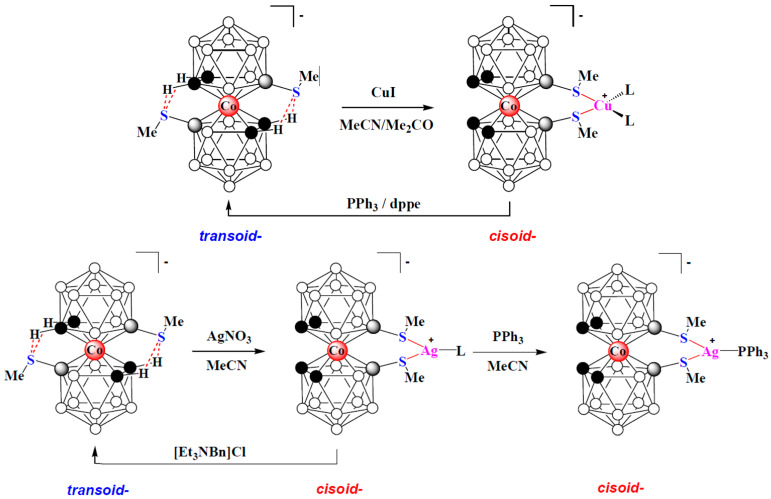
Reversible complexation of copper and silver with 8,8′-bis(methylsulfanyl) derivative of cobalt bis(dicarbollide).

More recently, the 8,8′-bis(methylsulfanyl) derivatives of cobalt, iron, and nickel bis(dicarbollides) [8,8′-(MeS)_2_-3,3′-M(1,2-C_2_B_9_H_10_)_2_]^−^ (M = Co, Fe, Ni) were proposed as organometallic modules for complexation-driven molecular switches [150,151]. These derivatives adopt the *transoid* conformation with the dicarbollide ligands rotated 180° relative to each other due to the formation of the intramolecular CH··· S hydrogen bonds between the ligands [109,110,111]. As in the case of the 1,1′-bis(diphenylphosphine) derivative of cobalt bis(dicarbollide), in the presence of external metal complexing agents, these weak intramolecular bonds are broken to form stronger S—>M donor–acceptor bonds with the metal ion. This process is well monitored using ^1^H and ^11^B NMR spectroscopy, which makes it possible to clearly distinguish between the coordination of a metal with one or two sulfur atoms [152]. If there are two free sites in the coordination sphere of the metal, it should lead to the formation of a chelate complex with a change in the conformation of cobalt bis(dicarbollide) from the *transoid* to the *cisoid* one. In turn, since thioesters are weak ligands, the addition of a stronger ligand should lead to the release of the 8,8′-bis(methylsulfanyl) derivative from the coordination sphere of the metal with their reverse transition from the *cisoid* to the *transoid* conformation. Indeed, such reversible transformations are observed when the 8,8′-bis(methylsulfanyl) derivative of cobalt bis(dicarbollide) reacts with copper and silver salts (Figure 37) [153].

**Figure 38:**
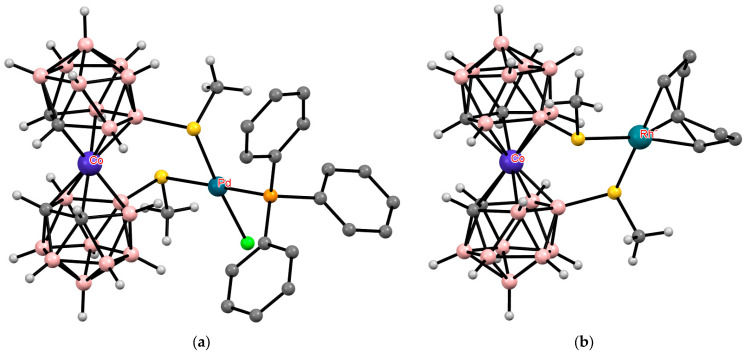
The solid-state structures of {(Ph_3_P)ClPd[8,9′-(MeS)_2_-3,3′-Co(1,2-C_2_B_9_H_10_)_2_]} (**a**) and {(COD)Rh[8,8′-(MeS)_2_-3,3′-Co(1,2-C_2_B_9_H_10_)_2_]} (**b**). Hydrogen atoms of organic ligands are omitted for clarity.

Other transition metals, such as palladium or rhodium, can also act as external complexing agents, complexation with which leads to the stabilization of the *cisoid* conformation of transition metal bis(dicarbollide) complexes (Figure 38) [153].

Thus, targeted changes in the conformation of transition metal bis(dicarbollide) complexes due to the breaking of one bond and the formation of others between dicarbollide ligands can be used in the design of molecular switches and sensors.

## 9. Conclusions

Growing interest in bis(dicarbollide) transition metal complexes is due to the use of their cyclic oxonium derivatives in the synthesis of boron-containing biologically active molecules. As for interligand interactions in derivatives of these complexes, until recently they did not attract much attention because there was no understanding of the effect they have on the properties of the compounds. Basic information about these interactions is based on X-ray diffraction data. Indirect data on the presence of such interactions can also be obtained using NMR spectroscopy and several other methods. The main contribution to these studies was made by research groups from the Institute of Inorganic Chemistry of the Czech Academy of Sciences, the Institute of Materials Science of Barcelona (ICMAB-CSIC), and the Nesmeyanov Institute of Organoelement Compounds of the Russian Academy of Sciences. As a result, the nature of interligand interactions and their influence on the properties of bis(dicarbollide) complexes have become a little clearer, but much remains to be achieved in this direction.

## Figures and Tables

**Table 1:** Translocation rate constants for cobalt bis(dicarbollide) and its 8,8′-dihalogen derivatives.

Compound	Translocation Rate Constant *k*, s^−1^
[3,3′-Co(1,2-C_2_B_9_H_11_)_2_]^−^	580
[8,8′-F2-3,3′-Co(1,2-C_2_B_9_H_10_)^2^]^−^	410
[8,8′-Cl_2_-3,3′-Co(1,2-C_2_B_9_H_10_)_2_]^−^	7430
[8,8′-Br_2_-3,3′-Co(1,2-C_2_B_9_H_10_)_2_]^−^	10,900
[8,8′-I_2_-3,3′-Co(1,2-C_2_B_9_H_10_)_2_]^−^	15,000

**Table 2:** Chemical shifts of *CH* groups in the ^1^H NMR spectra of cobalt bis(dicarbollide) and its 8,8′-dihalogen derivatives [8,8′-X_2_-3,3′-Co(1,2-C_2_B_9_H_10_)_2_]^−^ (X = H, F, Cl, Br, I).

X	CDCl_3_	CD_2_Cl_2_	Acetone-*d*_6_	DMSO-*d*_6_	Methanol-*d*_4_
H	3.76	3.84	3.98	3.99	3.86
F	3.71	3.79	3.90	3.88	3.75
Cl	4.34	4.35	4.28	4.30	4.21
Br	4.36	4.40	4.37	4.35	4.30
I	4.42	4.46	4.40	4.40	4.34
